# Splicing modulators act at the branch point adenosine binding pocket defined by the PHF5A–SF3b complex

**DOI:** 10.1038/ncomms15522

**Published:** 2017-05-25

**Authors:** Teng Teng, Jennifer HC Tsai, Xiaoling Puyang, Michael Seiler, Shouyong Peng, Sudeep Prajapati, Daniel Aird, Silvia Buonamici, Benjamin Caleb, Betty Chan, Laura Corson, Jacob Feala, Peter Fekkes, Baudouin Gerard, Craig Karr, Manav Korpal, Xiang Liu, Jason T. Lowe, Yoshiharu Mizui, James Palacino, Eunice Park, Peter G. Smith, Vanitha Subramanian, Zhenhua Jeremy Wu, Jian Zou, Lihua Yu, Agustin Chicas, Markus Warmuth, Nicholas Larsen, Ping Zhu

**Affiliations:** 1 H3 Biomedicine Inc., 300 Technology Sq, 5th Floor, Cambridge, Massachusetts 02139, USA

## Abstract

Pladienolide, herboxidiene and spliceostatin have been identified as splicing modulators that target SF3B1 in the SF3b subcomplex. Here we report that PHF5A, another component of this subcomplex, is also targeted by these compounds. Mutations in PHF5A-Y36, SF3B1-K1071, SF3B1-R1074 and SF3B1-V1078 confer resistance to these modulators, suggesting a common interaction site. RNA-seq analysis reveals that PHF5A-Y36C has minimal effect on basal splicing but inhibits the global action of splicing modulators. Moreover, PHF5A-Y36C alters splicing modulator-induced intron-retention/exon-skipping profile, which correlates with the differential GC content between adjacent introns and exons. We determine the crystal structure of human PHF5A demonstrating that Y36 is located on a highly conserved surface. Analysis of the cryo-EM spliceosome B^act^ complex shows that the resistance mutations cluster in a pocket surrounding the branch point adenosine, suggesting a competitive mode of action. Collectively, we propose that PHF5A–SF3B1 forms a central node for binding to these splicing modulators.

## Abstract (web summary)

A number of natural occurring small-molecule splicing modulators are known. Here, the authors combine chemogenomic, structural and biochemical methods and show that these compounds also target the spliceosome-associated protein PHF5A and propose a potential modulator binding site in the PHF5A–SF3B1 complex.

RNA splicing is catalysed by the spliceosome, a dynamic multiprotein–RNA complex composed of five small nuclear RNAs (snRNAs U1, U2, U4, U5 and U6) and associated proteins. The spliceosome assembles on pre-mRNAs to establish a dynamic cascade of multiple RNA and protein interactions that catalyse excision of the introns and ligation of exons^1^. Accumulating evidence has linked human diseases to dysregulation in RNA splicing, e.g., mutations in splicing regulatory sequences that disrupt the splicing of specific genes or functional alterations of spliceosomal components that impact the splicing of many genes^2^. Recent studies have revealed that splicing factors such as SF3B1, U2AF1 and SRSF2 are frequently mutated in multiple haematological malignancies including chronic lymphocytic leukaemia and myelodysplastic syndromes^3^. Therefore, much effort has been devoted to developing splicing-modulating small-molecules or oligonucleotides as therapeutic approaches to treating these diseases. Some of these have been or are being tested in clinical trials for cancer and severe neuromuscular diseases^4^,^5^,^6^,^7^.

A number of small-molecule compounds have been identified to possess selective activities in modulating or inhibiting splicing; however, the direct targets and/or exact mechanism of action are still ambiguous. Using biotinylated chemical probes and photo-crosslinking, the SF3b complex was identified as a binding target for spliceostatin A^8^, pladienolide B^9^ and herboxidiene^10^ (Supplementary Fig. 1). The SF3b complex is part of the U2 snRNA–protein complex (snRNP) assembled by U2 snRNA, splicing factors SF3a and SF3b, and other associated proteins. Together, these form the 17S U2 snRNP that assembles in an ATP-dependent fashion at the 3′ side of the intron to form the A complex^3^. The SF3b core complex contains several spliceosome-associated proteins (SAPs), including SF3B1/SAP155, SF3B2/SAP145, SF3B3/SAP130, SF3B4/SAP49, SF3B5/SAP10, SF3B6/SAP14a and PHF5A/SAP14b. These proteins are thought to bind to the branch point region. The most characterized target of small-molecule splicing modulators is SF3B1, which has been co-immunoprecipitated by biotinylated spliceostatin A^8^ and crosslinked to herboxidiene analogue^10^.

In addition to probing the physical interaction, phenotypic-resistant clone profiling has been utilized as a powerful method to uncover the cellular targets for small-molecule inhibitors, based on the hypothesis that under selection pressure, resistant mutation(s) in the target(s) or relevant pathway(s) are likely to offer the most survival advantage. This approach can also be used prospectively to predict resistance mutations that may emerge in a clinical setting^11^ and typically involves either stepwise induction of compound dosage or multiple rounds of enrichment to select for the most resistant clones^12^,^13^,^14^. Indeed, such an approach led to the discovery of a single amino acid substitution (R1074H) in SF3B1, which almost completely abolished the splicing-modulating and antiproliferative effects of pladienolide B and E7107 (ref. 12). This established that SF3B1 is likely a direct binding partner for pladienolides. However, the precise compound binding site and the role of other components of the SF3b complex remain unclear.

Here, by integrating structural and biochemical approaches with a modified chemogenomic strategy utilizing structurally different splicing modulators at permissive dosage for resistant mutation(s) mapping, we report that PHF5A, a core component of the SF3b complex, is a common cellular target of splicing-modulating chemical probes, including herboxidiene, pladienolide, spliceostatin and sudemycin. We also show that PHF5A-Y36, SF3B1-V1078 and SF3B1-K1071, in addition to previously reported SF3B1-R1074 (ref. 12), are key residues for small-molecule splicing modulators to bind to the SF3b complex. These mutations confer resistance to splicing modulation and cell growth inhibition induced by these compounds. Mechanistic investigation on PHF5A-Y36C reveals a dynamic splicing modulation (exon skipping (ES)–intron retention (IR) switch) by small-molecule splicing modulators, which reflects the differential sensitivity of individual introns to splicing modulator activities. Analysis of our crystal structure of human PHF5A and the yeast cryo-EM structure of the spliceosome B^act^ complex^15^ place these mutations at the interface of PHF5A and SF3B1 in the branchpoint adenosine (BPA) binding site. Biochemical analysis with reconstituted splicing modulator-binding protein complex and mutagenesis data of the Y36 residue further confirm the mechanism of the resistance mutation. Taken together, we propose PHF5A–SF3B1 form a binding site for these small-molecule splicing modulators, offering approaches to modulate specific splicing events.

## Results

### Splicing modulator-resistant mutations in PHF5A and SF3B1

**Figure 1:**
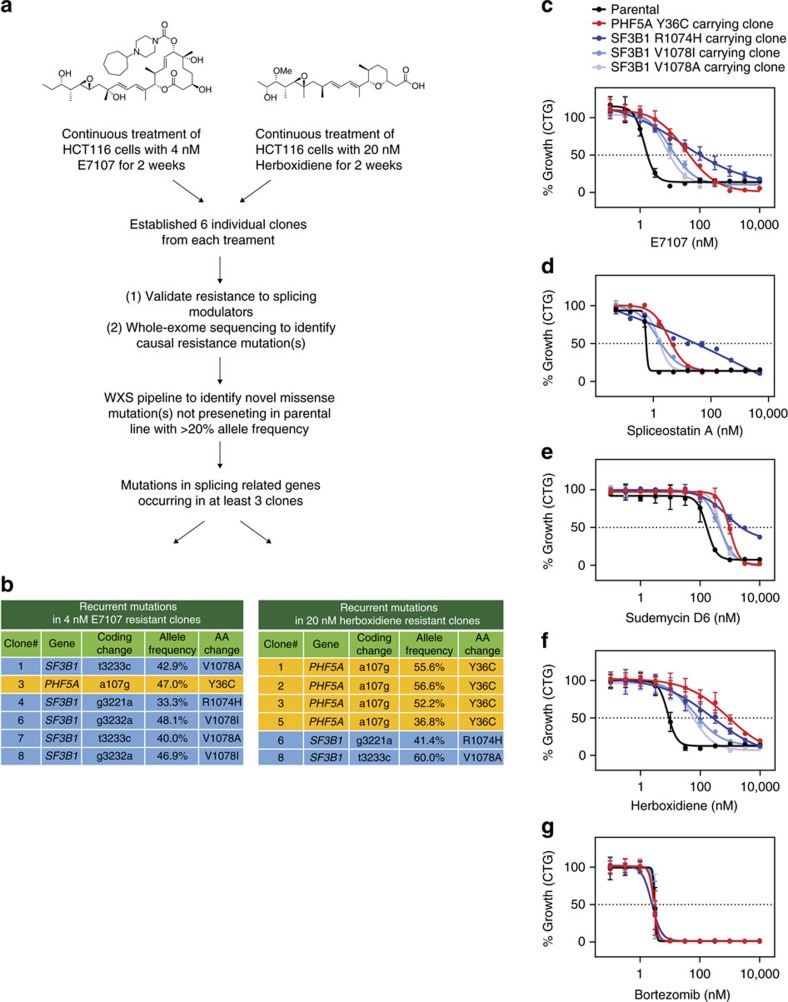
Recurrent splicing modulator-resistant mutations in PHF5A and SF3B1 identified by a chemogenomic approach. (**a**) Experimental scheme of E7107 and herboxidiene-resistant clone generation and whole-exome sequencing (WXS) analysis. (**b**) Recurrent mutations in E7107 and herboxidiene-resistant clones. (**c**–**g**) Seventy-two hours growth inhibition profiling (CellTiter-Glo cellular viability assay) of representative resistant clones' response to indicated compounds. Error bar indicates s.d. For E7107, herboxidiene and bortezomib, *n*=4; for spliceostatin A and sudemycin D6, *n*=2.

A group of splicing modulators were first discovered as natural products that are cytotoxic to cancer cells and later shown to target the SF3b complex^8^,^9^,^10^. To further investigate their mechanism of action, we explored the possibility of resistant clone generation with lower stress levels of compound via continuous administration of either lower dosage of E7107 (4 nM, ∼3 × GI_50_), a pladienolide derivative, or a less potent, structurally different splicing modulator, herboxidiene at 20 nM (∼3 × GI_50_) in HCT116 cells (Fig. 1a), whereas previous approaches used stepwise induction of pladienolide B or E7107 doses up to 100 nM (∼130 × GI_50_ in WiDR cells) to isolate resistant clones^12^. This less stringent approach could potentially mitigate off-target activity at high concentrations, as well as enhance the possibility to identify subtle but common mechanisms of splicing modulators. After 2 weeks of selection, six resistant clones from each treatment were expanded and subjected to whole-exome sequencing (WXS) to identify candidate causal genes for resistance to splicing modulators. Compared to the parental line, totally about 11,000 single-nucleotide variants and indels were identified with greater than 20% allele frequency (Supplementary Data 1). However, after cross-referencing with our curated splicing related gene list (Supplementary Data 2) and focusing on genes affected in at least three individual clones, mutations in only two genes were consistently scored. Five out of six E7107-resistant clones and two of the six herboxidiene-resistant clones carried mutations in SF3B1 (Fig. 1b), including the previously identified R1074H mutation and two previously unidentified mutations, V1078A and V1078I, strengthening the evidence that this region of SF3B1 is critical for splicing modulator action. Interestingly, the remaining E7107-resistant clone and four herboxidiene-resistant clones carried a Y36C mutation in PHF5A, an essential component of the SF3b complex (Fig. 1b). All identified mutations in PHF5A and SF3B1 were further confirmed by targeted Sanger sequencing (Supplementary Fig. 2). In addition, Sanger sequencing revealed that one independent clone from 20 nM herboxidiene treatment appeared to be a pool of two individual populations, which harboured both PHF5A-Y36C and a K1071E mutation in SF3B1 (Supplementary Fig. 2). While the apparent bias in mutation occurrences in either SF3B1 or PHF5A in the resistant clones (Fig. 1b) may implicate differences in how the pladienolide and herboxidiene scaffolds interact with the SF3b complex, these data strongly suggest that both proteins are common cellular targets for splicing modulators.

Growth inhibition profiling of the different resistant clones revealed that the SF3B1-R1074H mutation conferred the most robust resistance to E7107 whereas the PHF5A-Y36C and SF3B1-V1078 mutations were weaker (Fig. 1c). Interestingly, the SF3B1-R1074H mutation also conferred better resistance to spliceostatin A and sudemycin D6, both chemically related to FR901464, which is structurally different from pladienolides (Fig. 1d,e). In contrast, the PHF5A-Y36C mutation rendered more resistance in response to herboxidiene treatment (Fig. 1f), in line with the higher percentage of clones harbouring this mutation after herboxidiene selection (Fig. 1b). Mutations in SF3B1 or PHF5A did not affect the cell lines' sensitivity to bortezomib, a pan-cytotoxic proteasome inhibitor, highlighting the specificity of the mutations toward splicing modulators (Fig. 1g). To validate the apparent preference for different scaffolds, we expanded growth inhibition profiling to additional compounds and directly compared the GI_50_ shift in the SF3B1 R1074H clone over the parental line versus the GI_50_ shift in the PHF5A Y36C clone. It is evident that both resistant mutations conferred resistance to all examined splicing modulators. More importantly, compounds appeared to cluster based on their scaffold, with PHF5A Y36C showing better resistance to the herboxidiene analogues and SF3B1 R1074H showing better resistance to the pladienolide and spliceostatin analogues (Supplementary Fig. 3).

### PHF5A-Y36C confers resistance to splicing modulators

**Figure 2:**
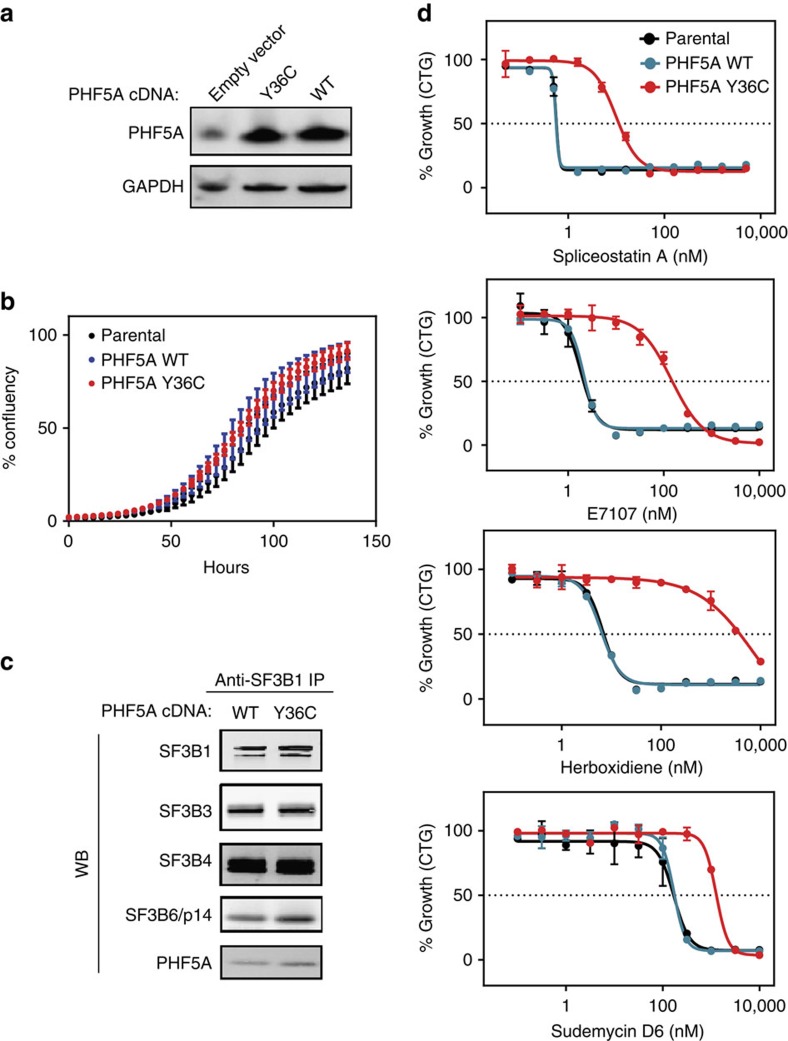
PHF5A-Y36C does not affect basal cellular functions but confers resistance to splicing modulators. (**a**) Western blot analysis of PHF5A levels in parental, PHF5A WT-expressing and PHF5A Y36C-expressing HCT116 cells. GAPDH is shown as a loading control. (**b**) Proliferation of parental, WT PHF5A-expressing or Y36C PHF5A-expressing HCT116 cells as measured by the Incucyte imaging system. *X* axis indicates hours post seeding, and *y* axis indicates percent of confluency. Error bar indicates s.d., *n*=5. (**c**) Western blot analysis of indicated SF3b complex protein levels following anti-SF3B1 pull-down from nuclear extracts containing WT or Y36C PHF5A. (**d**) Seventy-two hours growth inhibition profiling (CellTiter-Glo cellular viability assay) of parental, PHF5A WT-expressing and PHF5A Y36C-expressing HCT116 cells in response to indicated splicing modulators. Error bar indicates s.d., *n*=2.

To further validate PHF5A-Y36C as a mechanism underlying resistance to splicing modulation, we expressed either wild-type (WT) PHF5A or Y36C PHF5A at similar levels in the parental HCT116 cell line (Fig. 2a). Despite the sequence conservation of this tyrosine residue through evolution^16^, expression of either PHF5A-WT or Y36C has no apparent effect on cell growth (Fig. 2b), localization of SF3B1 protein or formation of nuclear speckles (Supplementary Fig. 4). Given that PHF5A is one of seven proteins in the SF3b complex, we then examined if the mutation could disrupt interactions with any of the core components and alter the overall composition of the complex. Immunoprecipitated (IP'ed) samples by anti-SF3B1 antibodies from WT and mutant cell lines were subjected to western blot and mass-spectrometry analysis to qualitatively assess their composition (Fig. 2c and Supplementary Fig. 5). We did not observe any significant differences in the overall composition of the complexes containing WT or Y36C PHF5A, suggesting that aside from this mutation they are otherwise intact and functional. Whole-transcriptome RNA-seq analysis confirmed that expression of PHF5A-Y36C accounted for ∼92% of the total *PHF5A* mRNA (Supplementary Fig. 6a) in the engineered cell line but had minimal effects on global splicing or gene expression when compared to WT (Supplementary Figs 6b and 8). Importantly, whereas parental cells and cells expressing WT PHF5A were sensitive to splicing modulator treatment, expression of PHF5A-Y36C conferred resistance to a panel of splicing modulators (Fig. 2d), phenocopying the spontaneous PHF5A Y36C resistant clones (Fig. 1c–f). This resistance phenotype appears to be general as it was also observed when PHF5A-Y36C was introduced to another cell line (Panc0504, Supplementary Fig. 7).

**Figure 3:**
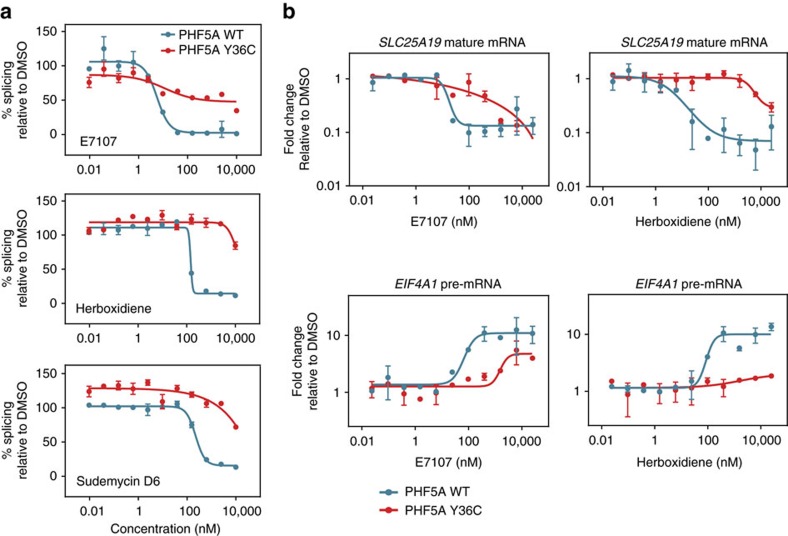
PHF5A-Y36C protects against splicing modulator induced mis-splicing. (**a**) *In vitro* splicing assay in the presence of indicated splicing modulators in nuclear extracts containing WT or Y36C PHF5A. Error bar indicates s.d., *n*=4. (**b**) Taqman gene expression analysis of mature *SLC25A19* mRNA levels and *EIF4A1* pre-mRNA levels in either WT- or Y36C PHF5A-expressing cells treated with indicated splicing modulators. All data points were normalized to the corresponding DMSO-treated control samples and displayed in logarithmic scale on the *y* axis. Error bar indicates s.d., *n*=2.

We next examined the behaviour of the PHF5A-Y36C mutation at the biochemical level. Consistent with the cellular data (Fig. 2d), *in vitro* splicing assays with an exogenous pre-mRNA substrate showed that the Y36C mutant protected against the inhibition by splicing modulators of different scaffolds (Fig. 3a). To validate whether similar levels of protection are also present *in vivo*, we used quantitative real-time PCR analysis to assay the splicing of two endogenous pharmacodynamic marker genes that were used previously in the phase I clinical trial of E7107 (ref. 5) (Fig. 3b). In agreement with the effect observed in *in vitro* splicing assays, Y36C mutation also reduced the inhibition on the production of spliced, mature *SLC25A19* mRNAs and the accumulation of unspliced, immature *EIF4A1* pre-mRNA elicited by splicing modulators (Fig. 3b).

### PHF5A-Y36C alters E7107 induced aberrant splicing

**Figure 4:**
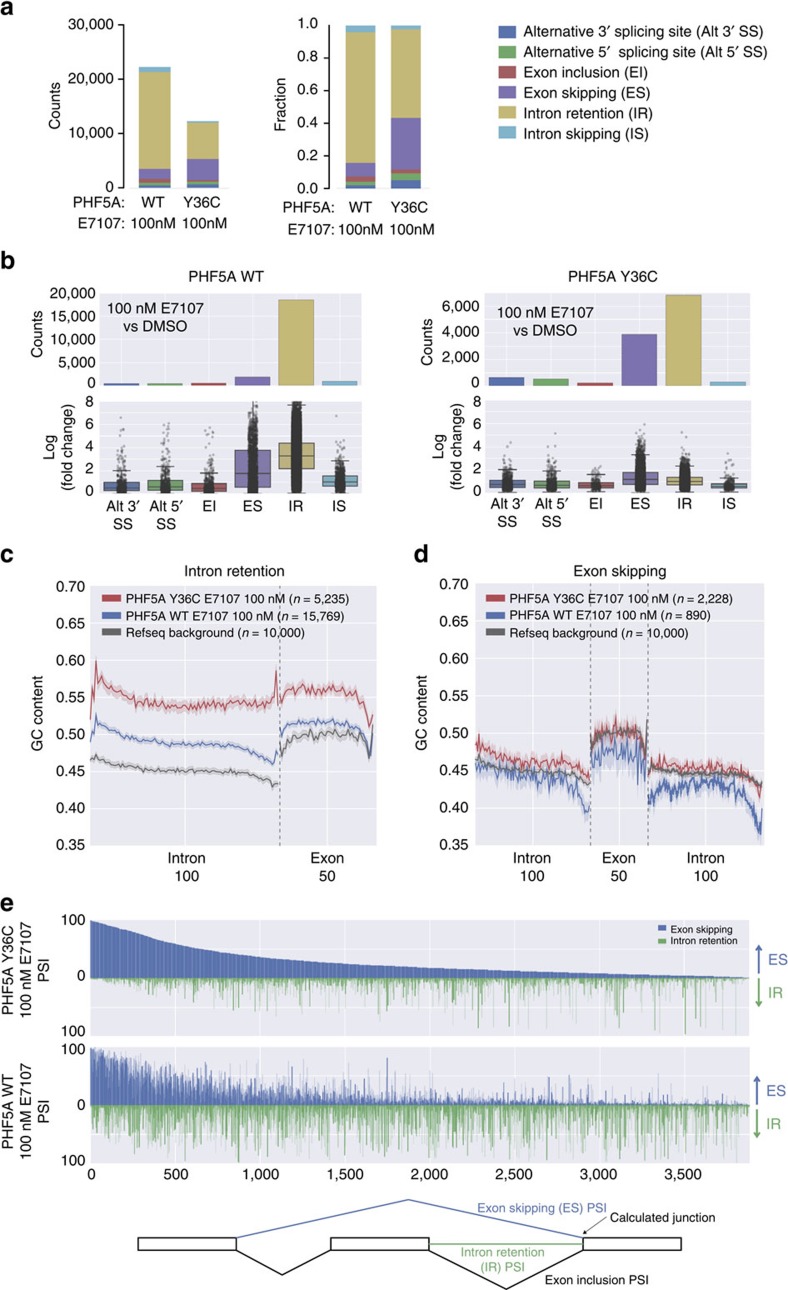
Inhibition and modulation of the effect of E7107 on global splicing patterns by PHF5A-Y36C. (**a**) Stacked bar graph of the counts (left panel) and fractions (right panel) of differential splicing events in each indicated treatment group as compared to DMSO controls. (**b**) Summary of the counts and log_2_ fold changes of differential splicing events in the indicated treatment group as compared to DMSO controls. Box shows the interquartile range (IQR) of the data set whereas the whiskers illustrate 1.5 × IQR. (**c**) Plot of average GC content within retained introns and downstream exons from E7107-induced intron-retention junctions. Each intron was normalized to 100 bins whereas each exon to 50 bins (see Methods for details). Dark line represents average GC content of each bin; shaded region indicates the 95% confidence interval. (**d**) Plot of average GC content within skipped-exons and both upstream (left) and downstream (right) introns from E7107-induced exon-skipping junctions. Each intron was normalized to 100 bins whereas each exon to 50 bins (see Methods for details). Dark line represents average GC content of each bin; shaded region indicates the 95% confidence interval. (**e**) Waterfall plot of the 3′ junction usage of 3,883 junctions (see text for details) in E7107 treated PHF5A Y36C (top) and WT (bottom) cells. *X* axis on both panels is ordered based on the ES PSI (percentage spliced in) value (large to small) of each junction in E7107-treated Y36C line. On *y* axis the PSI of either exon-skipping (ES, blue) or intron-retention (IR, green) of the same 3′ junction were shown. The scheme of PSI calculation is shown below waterfall plots.

To examine how global splicing is affected by splicing modulators, we applied whole-transcriptome RNA-seq analysis in both WT- and Y36C PHF5A-expressing cells treated with 100 nM E7107. Unsupervised clustering based on gene expression and principal component analysis of splicing junction usage confirmed that the Y36C cells treated with E7107 clustered away from their WT counterpart but near the DMSO-treated controls, suggesting that the Y36C mutation weakened E7107 activity as expected (Supplementary Fig. 8). Detailed differential splicing analysis further unveiled the quantitative and qualitative effects imposed by the Y36C mutation (Fig. 4a,b and Supplementary Data 3). Specifically, compared to the respective DMSO-treated controls, IR events were predominant in WT cells treated with E7107 as measured by both the number of events and average fold change (Fig. 4a,b, left bar). Consistent with the protective effect of Y36C, the overall amount of IR events and their average fold change were greatly reduced in the mutant cells treated with E7107 (Fig. 4a,b, right bar). Surprisingly, the number of compound-induced ES events was increased in the mutant cells compared to WT upon E7107 treatment (Fig. 4a,b), suggesting that PHF5A-Y36C-mediated resistance to splicing modulation involves a differential response at the global level.

The regulation of IR and ES events is known to be associated with exon/intron length and nucleotide content, as well as with specific chromatin marks^17^. Particularly, a differential in GC content between neighbouring introns and exons may have evolved as recognition signals for the splicing machinery^18^. Therefore, we sought to examine whether intronic GC content might also affect splice site recognition in PHF5A-WT or Y36C cells under splicing modulation (Fig. 4c,d). In WT cells, E7107-induced IR introns harbour higher GC content and less differential with the downstream exons as compared to the randomly selected background introns (Fig. 4c). Interestingly, IR introns/exons in PHF5A Y36C cells treated with E7107 displayed much higher GC composition and minimal differential between affected introns and exons as compared to its WT counterpart (Fig. 4c). In contrast, whereas ES junctions in compound treated WT cells showed lower GC composition than the background, ES junctions in Y36C cells treated with E7107 presented with higher GC content (Fig. 4d). In aggregate, these data suggest that intron/exon GC content may contribute to Y36C-mediated interference of splicing modulation.

Intriguingly, the intron/exon GC contents of IR events in WT cells (Fig. 4c, blue curve) are comparable to those of ES events in Y36C cells (Fig. 4d, red curve). In addition, E7107 treatment induced more ES events but fewer IR events in PHF5A-Y36C cells (Fig. 4a,b). Thus, we hypothesized that some of these ES-related introns from the Y36C cells might be switched to IR in the WT cells under the same E7107 treatment. To this end, we calculated the percentage (percent spliced in, PSI) of the individual 3′ intron–exon junction usage for these ES events in both PHF5A WT and Y36C cells. Theoretically, the outcome of these 3′ junctions would be either ES, IR or exon inclusion (for scheme of the calculation, see Fig. 4e and Methods). Consistent with our ES/IR switch hypothesis, 2,470 out of these 3,883 Y36C-related ES junctions (∼64%) showed reduced ES PSI and increase IR PSI in the WT cells treated with E7107 (Fig. 4e and Supplementary Data 4). This provided further evidence at the global level that PHF5A Y36C could weaken the activity of splicing inhibitors by modulating the usages of specific intron–exon junctions both quantitatively and qualitatively, utilizing the evolutionarily developed relative GC content of the neighbouring introns/exons^18^.

### PHF5A-Y36C alters E7107-induced IR-ES profile of *MCL1*

**Figure 5:**
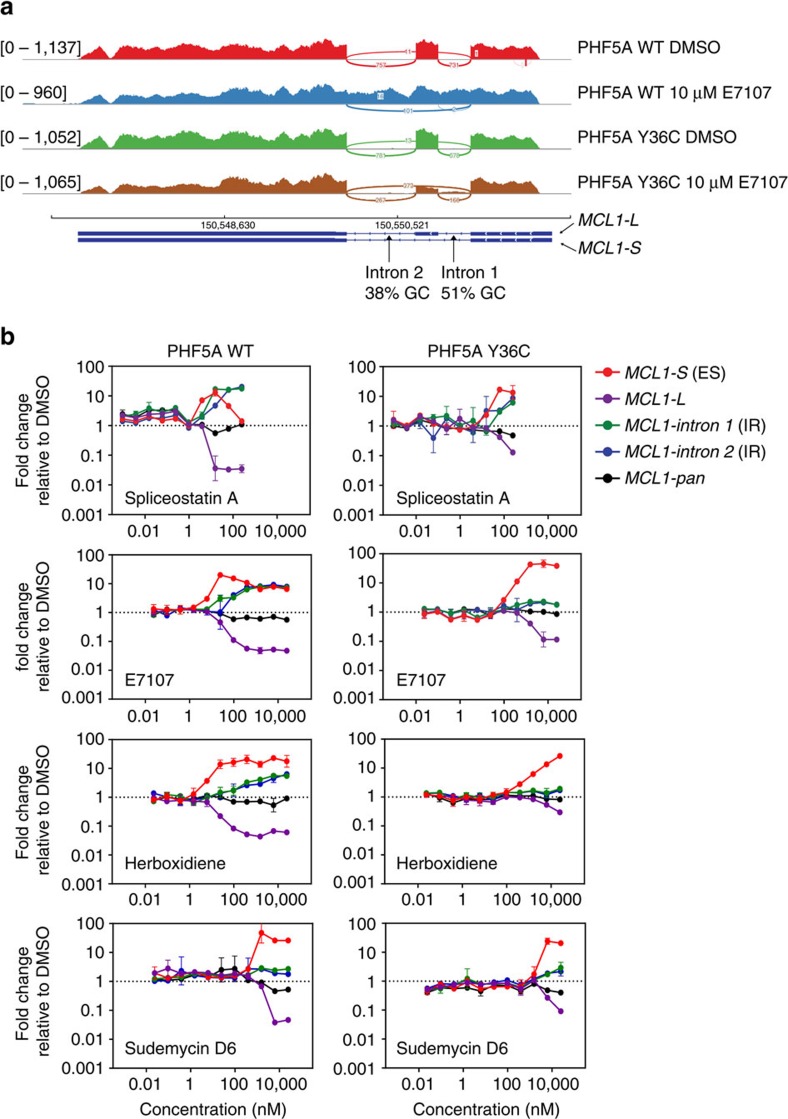
PHF5A-Y36C alters the effects of splicing modulators on *MCL1* splicing. (**a**) Representative Sashimi plot of the production of different *MCL1* isoforms under indicated treatment from either WT or Y36C PHF5A overexpressing cells. Total reads for each track are shown in the left. (**b**) Taqman gene expression analysis of indicated *MCL1* isoforms in either WT (left panel) or Y36C (right panel) PHF5A expressing cells treated with splicing modulators. Error bar indicates s.d., *n*=2.

We next focused on the specific genes modulated by E7107 in both genotype backgrounds. Despite differences in the number of splicing events elicited by E7107, the overall numbers of affected genes from WT or Y36 cells were comparable and shared a large overlap (Supplementary Fig. 9a and Supplementary Data 5). Gene Set Enrichment Analysis also identified candidate genes linked to essential pathways in either WT- or Y36C-specific genes (Supplementary Fig. 9b,c and Supplementary Data 5). To validate our global differential splicing analyses that revealed an IR/ES switch by splicing modulators in PHF5A-Y36C cells, we focused on genes that were associated with significant IR events in WT cells treated with E7107 as compared to DMSO controls, but were linked to significant ES events in Y36C under compound treatment (Supplementary Data 6). A large number of pivotal genes such as *MCL1*, *CDC25B*, *RBM5* and *CDK10* were among the group, and individual Sashimi plots validated the differential in splicing behaviour between WT and Y36C cells treated with E7107 (Fig. 5a and Supplementary Fig. 10). *MCL1* exists as two isoforms, *MCL1-L* and *MCL1-S*, and was previously reported as a major target for splicing modulators such as meayamycin B^19^,^20^ and sudemycin D1 (ref. 21). Interestingly, the second intron of *MCL1* harbours a low (38%) GC content compared to the GC-rich (51%) upstream intron. Sashimi plots of the *MCL1* RNA-seq data confirmed that in DMSO-treated control samples, both ES and IR events occurred at very low levels in WT and Y36C cells, resulting in dominant production of the canonical *MCL1-L* form (Fig. 5a). Upon E7107 treatment, IR was the dominant event observed in WT cells. In contrast, upon PHF5A Y36C expression, the effect of E7107 was largely altered, and mainly ES events were observed yielding the *MCL1-S* form (Fig. 5a).

Next, we utilized *MCL1* as a biomarker to expand our analysis of the ES/IR switch to additional splicing modulators of different scaffolds and multiple dosages. Taqman gene expression not only confirmed the RNA-seq analysis but also revealed a correlation between the potency of splicing modulators and the relative rates of induction for ES and IR events. Specifically, in PHF5A WT cells, the more potent spliceostatin A (GI_50_=0.76 nM in HCT116) led to similar kinetics for dose-dependent induction of *MCL1* ES and IR events, whereas the slightly less potent E7107 (GI_50_=1.5 nM in HCT116) presented with ‘earlier' induction of *MCL1* ES events than IR events at lower doses. The weaker herboxidiene (GI_50_=7.6 nM in HCT116) showed an even more pronounced effect, and finally the IR events were not observed with the weakest compound tested, sudemycin D6 (GI_50_=149 nM in HCT116) (Fig. 5b, left panels). These data strengthened the observation that the low GC containing intron 2 of *MCL1* was more resistant to splicing modulation than the higher GC containing intron 1 in the same gene. Importantly, expression of the PHF5A Y36C mutation delayed or blocked the onset of the *MCL1* IR events in the presence of these splicing modulators (Fig. 5b, right panels). Interestingly, *MCL1-S* production, representing ES events, was enhanced to a higher level in PHF5A-Y36C cells compared to WT upon increasing dosage of E7107 (Fig. 5b, second row). Taken together, these data confirmed the observation that PHF5A Y36C controlled the switch between compound induced IR events and ES events.

### Crystal structure of human PHF5A

**Figure 6:**
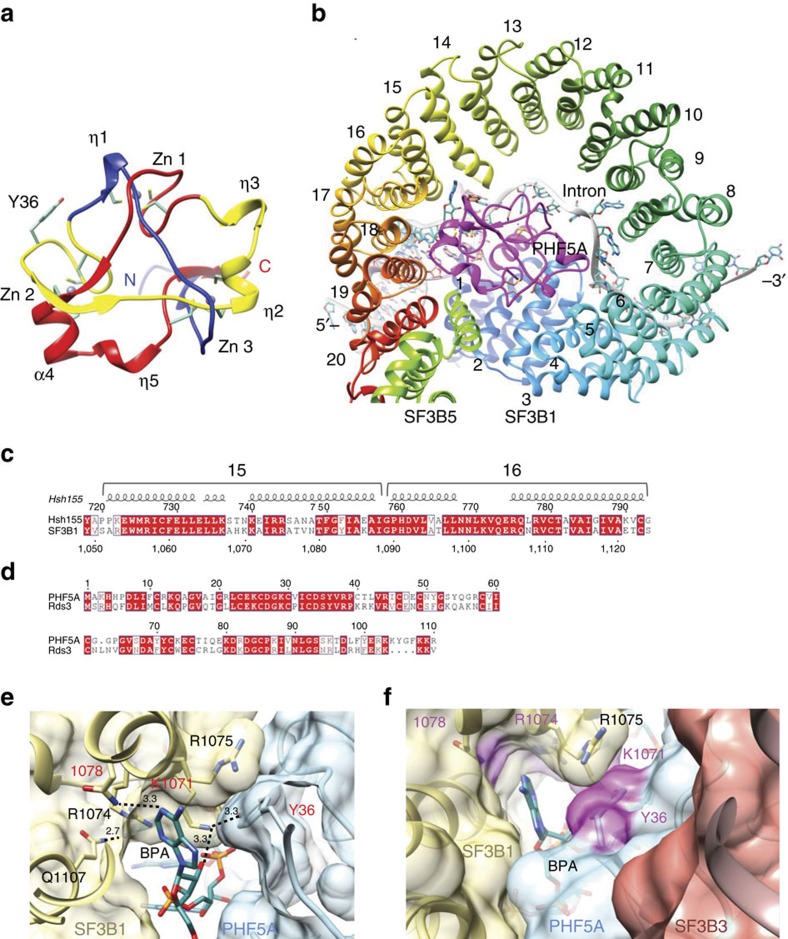
Crystal structure of human PHF5A. (**a**) Ribbon diagram of PHF5A (PDB:5SYB). Zinc atoms are shown as grey balls and form the vertices of a near equilateral triangle. The secondary structural elements (α: helix, η:310 helix, β: strand) forming the sides of the trefoil knot are coloured blue, yellow and red arranged by their primary sequence. The N and C termini are labelled. Cysteine residues are shown as sticks as well as the critical Y36 residue. (**b**) Model of PHF5A in the yeast B^act^ complex. Yeast PHF5A (magenta), SF3B5 (neon green) and SF3B1 (rainbow colours according to HEAT repeat HR-1 to 20) formed a complex that made contacts to the RNA duplex base-paired by U2 snRNA (orange ribbon) and the branch point sequence (BPS), and as well as a single-stranded intron RNA at the downstream of BPS (grey ribbon and the atoms are coloured in cyan). (**c**) Sequence alignment of the HEAT repeat 15 and 16 where this part of Hsh155 formed adenine-binding site with Rds3. (**d**) Sequence alignment of PHF5A with Rds3. The sequence identity is 56%. (**e**) Potential configuration of human adenine-binding site showing interactions between PHF5A (light blue), SF3B1 (yellow) and intron RNA (cyan). (**f**) Surface view of the potential modulator-binding site composed by SF3B1 (yellow), PHF5A (light blue) and SF3B3 (orange). Drug-resistant residues were highlighted in magenta.

**Figure 7:**
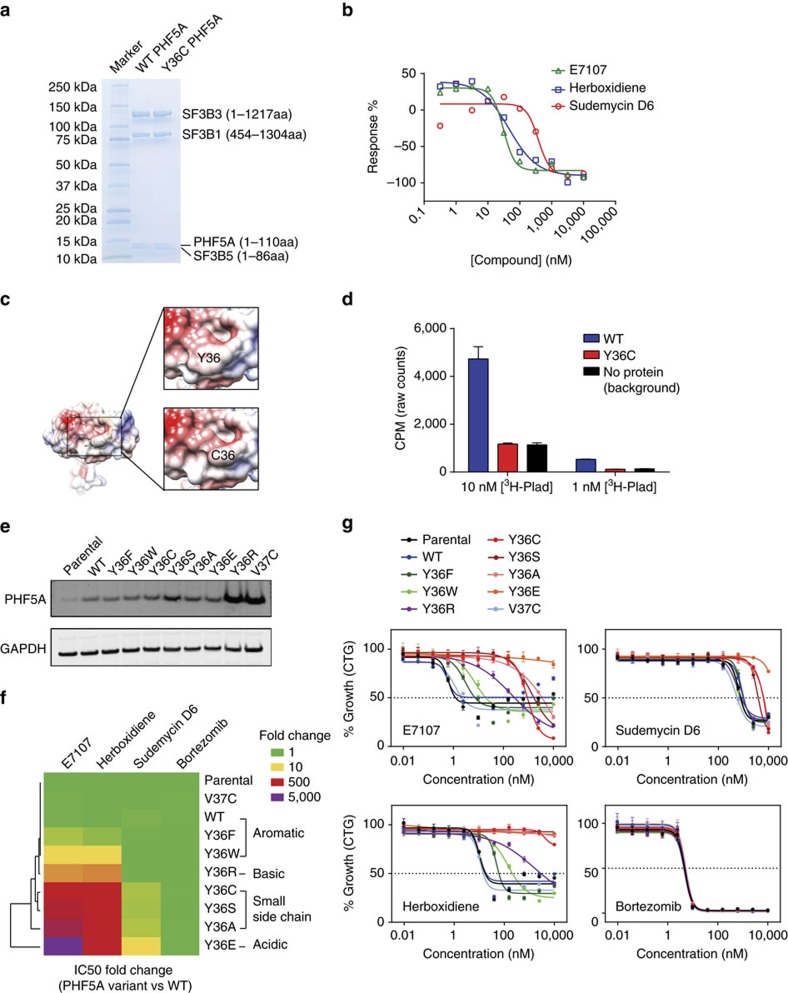
Characterization of the binding pocket of splicing modulator. (**a**) Coomassie staining of the recombinant four-protein mini-complexes containing PHF5A-WT or PHF5A-Y36C used for Scintillation Proximity Assays. (**b**) The competitive titration curves of non-radioactive splicing modulators to ^3^H-labelled pladienolide analogue (10 nM) binding to the WT four protein complex. (**c**) Overall surface view of modelled C36 overlaid onto WT (Y36 show in cyan stick) and zoom-in PHF5A surface view at Y36 and C36. Surface potential coloured in red: −8 kBT/e, blue: +8 kBT/e and white: 0 kBT/e was calculated by APBS. (**d**) Scintillation Proximity Assay of the ^3^H-labelled pladienolide analogue (10 and 1 nM) binding to protein complexes containing WT or Y36C PHF5A. Error bar indicates s.d., *n*=2. (**e**) Western blot analysis of PHF5A levels in parental and indicated PHF5A variants expressing HCT116 cells. GAPDH is shown as a loading control. (**f**) Unsupervised clustering heatmap of the IC_50_ shift between indicated PHF5A variant expressing cell lines as compared to WT cell lines. The shift is shown as fold changes and calculated from IC_50_ values extracted from dose–response curves in (**g**). Each row represents indicated PHF5A variant and each column corresponds to indicated compound. Colour key is shown on the top right corner. (**g**) Seventy-two hours growth inhibition profiling (CellTiter-Glo cellular viability assay) of parental and indicated PHF5A variant expressing HCT116 cells' response to indicated compounds. Error bar indicates s.d., *n*=3.

Given that Y36C PHF5A has no effect on basal splicing but plays a critical role in hindering and altering splicing modulators' effect on RNA splicing, we sought to explain the role of PHF5A in the context of the three-dimensional structure. We purified the WT protein and determined the crystal structure at 1.8 Å resolution (Table 1). Our final model contains residues 2–93 out of 110 total. PHF5A forms a mushroom-like structure with a triangular-shaped cap and a stem composed of antiparallel strands from the N and C termini (Fig. 6a and Supplementary Fig. 11d). The cap is formed by a left-handed, triangular, deep trefoil knot containing three zinc ions and five CXXC motifs, which are permuted between the zinc fingers (Supplementary Fig. 11b). PHF5A contains 13 Cys residues and 12 of these coordinate 3 zinc ions in tetrahedral geometry. The remaining cysteine was mutated to serine (C40S) to enhance soluble protein expression. Interestingly, PHF5A incorporates three different types of zinc finger. Zinc-finger 1 (ZnF1) folds into a gag knuckle and has C4 coordination from the first and fourth CXXC motifs. The first of these has a short helical turn (η1) while the fourth has a zinc knuckle^22^. Zinc-finger 2 (ZnF2) is formed by the second and fifth CXXC motifs. The first of these motifs is a zinc knuckle and the second comes from helix-α4 and therefore resembles the treble clef GATA-like zinc finger^23^. Zinc-finger 3 (ZnF3) is formed by the third CXXC motif from helix-η2 and two individual cysteines from the loops connecting the first and the last beta strands of the mushroom stem. This third zinc finger resembles an interrupted classical ββα finger with a short helix^16^,^23^. Given the location of PHF5A-Y36 on the surface near the second zinc finger, and the evidence that it does not alter any tested cellular activities, we would predict that mutation to Cys would have minimal effect on the overall fold but rather act locally altering the surface topology (Fig. 7c).

While classified as a PHD finger, PHF5A has low sequence homology with other PHD fingers and differs from the canonical fold. A high level of sequence identity across diverse eukaryotic organisms shows its unique trefoil knot topology is likely to be conserved (Fig. 6d and Supplementary Fig. 11a). At the same time, PHF5A has very low sequence identity when compared to other sequences within the same organism, suggesting a unique biological role in the cell. However, proteins with low sequence identity can still share similar three-dimensional structures and have similar function. To explore this possibility, we compared our structure to all other available structures in the PDB and found only one other protein with similar fold, Rds3, a PHF5A homolog from yeast^24^. The Rds3 structure was solved by NMR, containing 80 residues and unstructured coils at the N- and C- termini^16^. It also has three zinc fingers and the same trefoil knot fold (*Z*-scores 12.6 and RMSD 2.2 Å)^24^.

The full-length Rds3 protein was recently observed in the cryo-EM structure of the spliceosome B^act^ complex at a resolution range of 3.0–3.5 Å^15^. This structure shows that Rds3/PHF5A is a central scaffolding protein, interacting with Hsh155/SF3B1, Rse1/SF3B3, Ysf3/SF3B5, U2 snRNA and the intron RNA (Fig. 6b). Here, the SF3B1 HEAT repeats (HR) form a right-handed superhelical spiral of one complete turn forming a central ellipsoid cavity of approximately 34 × 39 Å (Fig. 6b). PHF5A nestles into this cavity forming extensive contacts along its sides with HR 2–3, 6, 15 and 17–20 (Fig. 6b). Of 110 total residues in PHF5A, 28 are forming contacts with SF3B1 burying 19% (1,337 Å^2^) of surface area and a high degree of sequence conservation between the two interfaces. The C-terminal HR-20 helix and N-terminal helix of SF3B5 form a parallel helix–helix interaction that completes the superhelical turn while forming additional interactions with PHF5A (residues F6-L12) (Fig. 6b). SF3B3 sits along the top face of the SF3B1–PHF5A complex forming contacts with both, while the intronic RNA sits along the bottom face of the complex. Most of these interactions are to the phosphodiester backbone, as evidenced by complementary electropositive surface (Supplementary Fig. 11c).

Superimposing the yeast and human PHF5A structures reveals structural differences at only two regions, which both form interactions with the intron RNA (Supplementary Fig. 11d). The last helix (G93-R110) of the C-terminus, which is missing in the PHF5A crystal structure, contains conserved basic residues located between HR-2 from SF3B1 and the intron-U2 RNA duplex. These basic residues form multiple contacts to the intron nucleotides (+1-CACAUU) (Supplementary Fig. 11d) downstream of BPA (position 0). A minor difference is at the helix (η2)–loop–helix (η3) (from N50-R57) near ZnF3 where it has lower sequence conservation and also adopts multiple conformations in the Rds3 solution structure, suggesting this part of the molecule might be flexible. This region is making contact to two nucleotides (+9-AU) from the intron and the flexibility could accommodate conformations of different intronic RNAs.

### Structural analysis of resistant mutations in PHF5A–SF3B1

Recently, several cryo-EM structures have provided snapshots of the pre-catalytic and catalytic steps in the splicing reaction. The SF3b complex was only observed in the pre-catalytic B^act^ complex^15^. In the next step, rearrangements occur triggering dissociation of the SF3b complex and formation of the C complex, in which the phosphodiester bond has been made between the 2′-OH of the BPA and the 3′ phosphate of guanosine at the 5′-splice site^25^,^26^,^27^. Strikingly, the yeast B^act^ complex cryo-EM structure shows that the interface between PHF5A and SF3B1 is where the BPA binds^15^ (Fig. 6e). These proteins from the SF3b complex apparently shield the reactive group from premature nucleophilic attack. Indeed, in this model, PHF5A-Y36 forms direct contacts with the BPA, clearly implicating PHF5A in branchpoint recognition. This specialized biological role may explain its high sequence conservation and lack of any other apparent counterparts in the cell, which is consistent with previous finding of its key roles in splicing regulation and splicing modulator sensitivity in glioblastoma stem cells^28^. The HEAT repeats of SF3B1 that define this binding pocket (HR15-17) are also highly conserved (Fig. 6c and Supplementary Fig. 12). Interestingly, the resistance mutations identified in this study, PHF5A-Y36C, SF3B1-K1071E, SF3B1-V1078A/I, and previously reported SF3B1-R1074H, all cluster around this pocket (Fig. 6e,f). Moreover, crosslinking data show that these splicing modulators interact directly with SF3B1 and SF3B3 (refs 9, 10), which sits immediately above this pocket (Fig. 6f). These striking coincidences provide strong evidence that this BPA-binding pocket is also the region where splicing modulators bind. While conferring resistance, remarkably these mutations are not detrimental to basal splicing despite their proximity to the BPA. Detailed analysis shows that SF3B1-K1071 is a conserved residue (Fig. 6c) and forms H-bonds with the 2′-hydroxyl of the BPA ribose sugar and also with the hydroxyl of PHF5A-Y36, which helps to position and orient these residues at the interface (Fig. 6e). Since mutation of either of these residues results in resistance, this interaction is likely important for modulator binding. PHF5A-Y36 also forms extensive van der Waals interaction with another conserved residue, SF3B1-R1075, which also helps orient this side chain and alter the binding pocket. Based on our Y36C model, the mutation does not cause a significant change to the electrostatic surface but does alter the surface topology (Fig. 7c). The loss in affinity suggests the aromatic side chain at this position is critical for splicing modulator binding. SF3B1-R1074H is located at the base of this binding pocket (Fig. 6e). It does not make any direct interactions with RNA or PHF5A, but mutation would alter the shape of the binding pocket and could affect compound binding but not BPA interaction (Fig. 6e,f). SF3B1-V1078A/I is near the top of this pocket and not conserved between yeast and human (Fig. 6c). In yeast, this residue forms an H-bond to the BPA adenosine, but in humans this residue is likely to result in a relatively subtle change and indeed confers the least amount of overall resistance.

### Reduced binding affinity of splicing modulator to PHF5A-Y36C

To demonstrate the splicing modulator binding site is at the interface composed by SF3B1, PHF5A and SF3B3, we engineered a recombinant protein complex based on the yeast B^act^ cryo-EM structure^15^. By co-expressing these three proteins with SF3B5, we were able to reconstitute a stable 250 kDa complex that could be purified in two steps (Fig. 7a). To validate this recombinant complex can recapitulate a functional modulator binding site, we captured it on scintillation proximity assay (SPA) beads and probed its interaction with a ^3^H-labelled pladienolide analogue^9^. SPA assays revealed ^3^H-labelled pladienolide probe bound to the complex and other non-radioactive splicing modulators were able to compete off the bound probe, demonstrating the specificity of the interaction (Fig. 7b). In this competition assay, reduced signal from titrating non-radioactive modulators reveals the relative affinity of these three compounds to the complex compared to the pladienolide-like analogue and is consistent to the potency and rank ordering seen in the *in vitro* splicing (IVS) assay (Fig. 3a) and the cellular assay (Fig. 2d). This validates that these four proteins reconstitute a functional binding site for splicing modulators.

Next, the corresponding complex containing PHF5A-Y36C was generated to inspect whether the observed resistance mutation is a result of reduced binding between splicing modulator(s) and the SF3b complex. The purified PHF5A-Y36C recombinant complex was captured on the SPA beads and the same ^3^H-labelled tracer compound^9^ was used to probe the interaction at two different concentrations, 10 and 1 nM. SPA assay reveals that an approximate five-fold induction of the 10 nM ^3^H-labelled probe binding to the WT PHF5A containing complex over background, whereas the binding to the PHF5A-Y36C complex was equal to background. This demonstrates that the single Y36C mutation is sufficient to reduce modulator binding significantly (Fig. 7d) and suggests Y36 makes critical interactions to modulators. The reduced affinity was also observed in the IP'ed SF3b complex from PHF5A-Y36C cell nuclear lysates, confirming that this mutation is able to decrease modulator binding in a physiological relevant protein complex as well (Supplementary Fig. 13).

### Mutagenesis shows Y36 is critical for modulator activity

Our structural and functional analysis implicates the importance of an aromatic side chain for splicing modulator binding (Fig. 7c). To further dissect the importance of this interaction, we generated cell lines expressing additional amino acid substitutions at the Y36 and neighbouring V37 position (Fig. 7e–g). Western blot analysis confirmed comparable expression levels of most exogenous PHF5A variants (Fig. 7e). We then examined the levels of cell growth inhibition elicited by splicing modulators in these lines (Fig. 7f–g). Consistent with our hypothesis, substitution of Y36 with either phenylalanine (F) or tryptophan (W) only conferred a very minor protective effect against splicing modulators (Fig. 7g, dark and light green lines). Indeed, unsupervised clustering of the IC_50_ shift (PHF5A variant over WT cells) grouped WT cell lines and Y36F/W cell lines together (Fig. 7f). In contrast, when Y36 was mutated to either serine (S) or alanine (A), mimicking the smaller size of cysteine (C), strong resistance to splicing modulators was observed (Fig. 7g, dark and light red lines), and Y36S/A clustered closely with the Y36C-expressing cell line (Fig. 7f). Interestingly, introducing a charged amino acid at this position also reduces compound action. Specifically, when Y36 was mutated to glutamic acid (E), a bulky residue with negative charge, almost complete resistance to splicing modulators was observed (Fig. 7g, orange lines and Fig. 7f). Whereas when the residue was mutated to the positive charged arginine (R), albeit at a much higher expression level than other variants (Fig. 7e), the level of resistance was milder (Fig. 7g, purple lines and Fig. 7f). Taken together, this suggests that the aromatic group is required for binding and smaller residues or charged residues reduce modulator binding. In contrast, substitution of the V37 residue with cysteine (C) did not provide any protection against splicing modulators even at high expression levels (Fig. 7g, light blue lines and Fig. 7e–g). Consistent with previous data, the resistance only occurred to the treatment with splicing modulators but not bortezomib (Fig. 7f,g). Importantly, for all Y36 variants tested, the bias between resistances to different splicing modulator scaffolds persists, suggesting that the Y36 residue is more critical for interaction with herboxidiene than for pladienolide and sudemycin, which highlights that these splicing modulators could adopt different poses within this common binding pocket (Fig. 7f,g).

## Discussion

**Figure 8:**
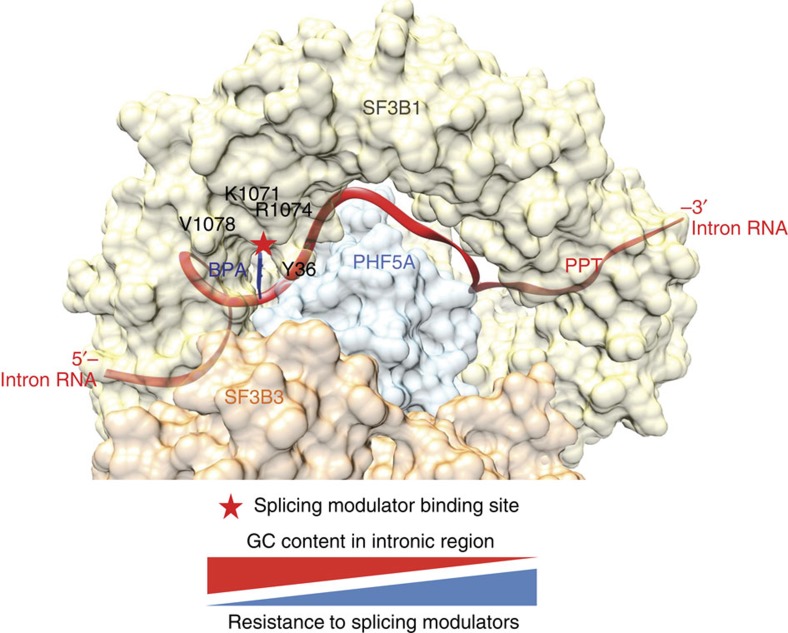
Model of splicing modulator interaction with the SF3b complex at the BPA-binding pocket constituted by PHF5A and SF3B1. The molecular surface representation of the protein complex SF3B1 (yellow), PHF5A (blue) and SF3B3 (orange). The intron RNA is shown as red ribbon, with branch point adenosine (BPA) in dark blue. The common splicing modulators binding site is indicated by a star with the approximate positions of the surrounding residues for which resistance mutations were identified. The figure was generated using the yeast B^act^ complex coordinates. The schematic model indicates the inverse correlation between the GC content of the intron sequence and their resistance to splicing modulation. Specifically, high GC content intron substrates are weaker substrates that show more sensitivity or less resistance to splicing modulators.

Spliceosomes undergo multiple ATP-dependent conformational changes involving a number of snRNPs, and this dynamic complexity makes it challenging to determine where and when splicing modulators bind. Previous photocrosslinking studies with pladienolide and herboxidiene analogues narrowed down the interaction point to the SF3b complex, one of the subunits of the U2 snRNP, specifically to the individual proteins SF3B3 and SF3B1 (refs 9, 10). The resistant mutation SF3B1-R1074H generated under high doses of pladienolide B and E7107 provided further evidence that SF3B1 is critical for compound binding^12^. By applying a genomic resistance mapping approach with low doses of E7107 and herboxidiene, we were able to elicit a number of different resistance mutations. This allows us to further refine the splicing modulator binding pocket and potentially to account for mechanism of action among certain introns. We uncovered a series of key mutations, Y36C in PHF5A, V1078A/I, K1071E and the previously identified R1074H^12^ in SF3B1, which, together with the reported photocrosslinking data^9^,^10^, allow us to pinpoint the modulator binding pocket to the interface between PHF5A and SF3B1 (Fig. 8). It remains possible that E7107 can act on additional regulators in the spliceosome subcomplex. The other two modulators, spliceostatin A and sudemycin D, also show resistance to the Y36C clone, indicating these compounds interact with this site as well^8^,^21^. Indeed, we confirmed the binding of splicing modulators to this common binding pocket by reconstituting a functional 4-protein complex consisting of PHF5A, SF3B1, SF3B3 and SF3B5 (Fig. 7a). The single amino acid substitution of Y36C reduced the binding of the pladienolide probe to background levels, suggesting that the mechanism of resistance is due to the decreased affinity of splicing modulators to the binding pocket (Fig. 7c). Detailed site-directed mutagenesis of Y36 shows that both the aromatic ring and electrical charge at the Y36 residue is critical for the activity of splicing modulators (Fig. 7e–g). Furthermore, mutations at Y36 revealed different levels of protection against these modulators with different scaffolds, indicating that these modulators may adopt slightly different poses within mode of interaction at this common binding pocket. Webb *et al*.^29^ have previously hypothesized several pharmacophore features for herboxidiene activity including a hydrophobic motif (a diene group) between C8 and C11. Pladienolide and herboxidiene share this diene moiety, implying this may bind at the proximity of Y36. Thus, it would be interesting to examine the relations between the aromatic ring of Y36 and this motif in the future.

Given the location of the resistance mutations around the BPA binding site, one possible model for the mechanism of action is that the splicing modulators are BPA competitive inhibitors (Fig. 8). This close proximity of splicing modulators binding pocket to the BPA is consistent with previous reports from the Valcarcel group and Reed group that both spliceostatins and pladienolides impair the canonical base pairing between U2 snRNA and pre-mRNA branch point region in the presence of heparin^25^,^30^. Collectively, these observations led to a model where splicing modulators directly impact on the fidelity of SF3B1 branch site recognition with consequences on the 3′ splice site recognition^30^. This competitive binding model immediately suggests several possible functional consequences that can be examined at the global splicing level. Specifically, weaker GC-rich intron substrates would be easier to inhibit than stronger intron sequences and this differential could manifest itself through alterations in splicing preferences in the presence of different compounds.

Consistent with this model for inhibition, we observed a nonlinear dose response in global splicing due to variations in individual intron ‘strength'. Splicing modulation is a global phenomenon which impacts more than 200,000 introns in the human genome^31^. Despite several conserved features within introns and adjacent exons, regulation of individual introns during splicing is both diverse and complex. This variation and complexity means that small-molecule inhibition will have differential effects on splice junction usage. Here, a protective mutation in PHF5A allowed us to examine the individual cellular responses of introns upon splicing modulation, which revealed transitions between IR and ES events.

It has been proposed that during evolution, the generally shorter, low GC containing introns in lower eukaryotes evolved under two different routes^18^: one group of introns remained short, but had markedly increased GC percentage and had less differential in term of GC composition compared to their neighbouring exons. Due to the shorter length of these introns, they are more likely to be recognized by an intron-defined splicing mechanism. Interestingly, these introns appear to be more susceptible to IR upon E7107 treatment. More importantly, we observed that when the effect of E7107 was weakened in the presence of PHF5A Y36C mutation, the average GC compositions of IR event-related introns were markedly higher with little to no differential from downstream exons (Fig. 4c). Given that the differential in GC composition between introns and surrounding exons might contribute to splicing machinery recognition, it is plausible to hypothesize that these kinds of introns are inherently more difficult for the splicing machinery to recognize, which in turn might make them easier to inhibit with splicing modulators. It has also been proposed that higher GC content around BPA may lead to a more stable secondary structure of the pre-mRNA; therefore, it is also plausible that GC content may affect the effectiveness of competition between pre-mRNAs and splicing modulators via structural and spatial mechanisms^32^.

In contrast, another group of introns maintained their low GC composition and large differential with adjacent exons during evolution, but underwent significant increases in length, which likely brought them out of the range of intron-defined splicing and converted them to an exon-defined splicing mechanism^18^. Intriguingly, under E7107 treatment, introns associated with increased ES events are associated with lower GC composition and higher GC differential with the skipped exons (Fig. 4d). Similar to the observation in IR events, the GC content of compound induced ES introns in the presence of Y36C was also higher than that of the WT cells (Fig. 4d). A higher differential in GC composition between introns and exons has been linked to increased nucleosome occupancy and enrichment of SF3B1 association with the chromatin, which presumably primes these junctions for co-transcriptional splicing^18^,^33^. Further characterization of the genomic structure of the junctions associated with ES events may yield additional insight in our understanding of the complex link between transcription and splicing.

Our observation that 2,470 junctions can be switched between IR and ES upon E7107 treatment depending on the genotype of PHF5A strengthens the hypothesis that introns possess differential sensitivity to small-molecule inhibitors (Fig. 4e). The fact that IR and ES events affect the same 3′ junction are not mutually exclusive further unveils the plasticity of splicing regulation and a fine-tuning mechanism of the usage of individual junctions (Fig. 4e). It is conceivable that in PHF5A WT cells, E7107 was efficient in competing with the canonical BPAs in these 2,470 junctions and led to IR events. However, upon PHF5A Y36C expression, E7107 would become less efficient in the competition with these junctions while maintaining its competence with the immediate upstream introns, which induced more ES events (Supplementary Fig. 14). Collectively, these differential sensitivities from cellular introns are consistent with the model that splicing modulators act as competitive BPA inhibitors, and are likely to result in the nonlinear response to differential dosages of splicing modulators.

Phenotypic screening of small-molecule libraries is a powerful way to identify potential drugs. However, cellular target identification for the screening hits has been an unremitting challenge. Several unbiased approaches have been developed to identify the cellular targets and mechanisms of action, including biochemical approaches such as affinity purification coupled with quantitative proteomics, genetic interaction approaches such as RNAi screening and domain-focused CRISPR screens, and computational inference approaches^34^,^35^. More recently, we and others used next-generation sequencing-based genomic or transcriptomic profiling of phenotypically resistant cell populations^13^,^14^,^36^ to identify unique recurrent single-nucleotide variations or expression alterations to illuminate potential cellular targets of compounds. Here, we further developed the method by screening structurally unrelated compounds at different low concentrations, in order to (1) mitigate the potential off-target activity at high concentrations and (2) enhance the possibility to identify subtle but common mechanisms of chemical probes. This allowed us to uncover multiple mutations/genes encoding proteins co-existing in the same complex. Interestingly, in our case, the finding of resistant mutations to PHF5A-Y36, SF3B1-V1078 and K1071, in addition to confirming the previously reported SF3B1-R1074, suggests the proximity of these residues to the action site of splicing modulators. The fact that corresponding amino acids of these residues in yeast were recently shown to form a pocket that accommodates the invariant adenosine in the BPS demonstrates that this genomic profiling strategy can provide faithful and informative insights into the action of candidate compounds. Hence, we propose that further expansion of the genomic profiling approach will offer a unique way to explore the MoA (mechanisms of action) for compounds using the ‘2-dimensional' genomic fingerprint dissection. This is particularly valuable when the protein structure and/or biochemical assays with purified proteins are not readily available as exemplified in this study by the complex and dynamic spliceosome.

In summary, we identify PHF5A as a key node of interaction for small-molecule splicing modulators. Our structural analysis pinpointed a common binding site around the branch point adenosine-binding pocket. Also, our results demonstrate how a single amino acid change on PHF5A Y36 weakened the inhibitory effect of splicing modulators and altered the global splicing pattern between ES events and IR events. We expect these findings will assist future development of more specific small-molecule splicing modulators for treating diseases associated with aberrant splicing.

## Methods

### Materials

Parental HCT116 cells were obtained from ATCC and cultured in RPMI 1,640 medium (Thermo Fisher, GIBCO#11875) supplemented with 10% fetal bovine serum (FBS). Parental Panc0504 cells were obtained from ATCC and cultured in GIBCO RPMI 1,640 medium (Thermo Fisher, GIBCO#11875) supplemented with glucose (to 4.5 g l^−1^ final), HEPES (10 mM final), sodium pyruvate (1 mM final), human insulin (10 μg ml^−1^ final) and 15% FBS. Cell line authentication was achieved by genetic profiling using polymorphic short tandem repeat (STR) loci (ATCC). All cell lines were free of mycoplasma contamination. Lenti-X-293T cells (Clontech Laboratories, Inc., Cat # 632180), a cell line for lentiviral packaging, was maintained in Dulbecco's modified Eagle's medium (Thermo Fisher, GIBCO#11965) containing 10% FBS and 4 mM L-glutamine. WT PHF5A cDNA in pShuttle vector was obtained from Genecopoeia (cat#GC-V1039) and cloned into pLenti6.3/V5 vector (Thermo Fisher) through LR recombination using Gateway LR Clonase II Enzyme Mix (Invitrogen). Mutagenesis of Y36 and V37 were carried using the Agilent Quickchange II kit following the manufacturer's recommendation using the PHF5A WT plasmid. All primers used for mutagenesis were designed using the QuickChange Primer Design tool by Agilent and listed in Supplementary Table 1. Verified positive clones of PHF5A Y36 or V37 variants were used for lentivirus production using X293T cells. Parental HCT116 cells and Panc0504 cells were then infected with virus containing medium and selected with Blasticidin S (Thermo Fisher) at 10 μg ml^−1^ for 1 week. Engineered cell lines were maintained in the same medium without antibiotics. The following primary antibodies were used at 1:1,000 dilution for western blot analysis in LI-COR buffer (LI-COR): α-SF3B1 mouse monoclonal antibody (MBL, D221-3), α-SF3B3 rabbit polyclonal antibody (Protein Tech, 14577-1-AP), α-SF3B4 goat polyclonal antibody (Santa Cruz, 14276), α-SF3B6/p14 rabbit polyclonal antibody (Protein Tech, 12379-1-AP), α-PHF5A rabbit polyclonal antibody (Protein Tech, 15554-1-AP). α-GAPDH rabbit polyclonal antibody (Sigma, G9545) was used at 1:10,000. Anti-rabbit and anti-goat IRDye-800CW secondary antibody (LI-COR) was used at 1:5,000 dilution and anti-mouse IRDye-680LT secondary antibody (LI-COR) was used at 1:20,000 dilution. Western blot was imaged using an Odyssey V3.0 imager (LI-COR). Uncropped images of western blots and gels are shown in Supplementary Fig. 15.

### Compounds

Bortezomib (PS-341) was purchased from LC Laboratories (Cat. No. B-1408, Lot: BBZ-112). E7107 and ^3^H-labelled Pladienolide probe were provided by Eisai Co. Ltd and their synthesis was previously reported^9^. Herboxidiene was also provided by Eisai Co. Ltd. Spliceostatin A and Sudemycin D6 were synthesized in house following established procedures^37^,^38^. For splicing modulators, the compound identity and purity was assessed by LC/MS and proton NMR (Supplementary Fig. 16). Purity was determined using a Waters H class Acquity ultra performance liquid chromatography system with an XSelect CSH C18, 1.7 μm 2.1 × 50 mm column, a flow rate of 0.8 ml min^−1^ at 20 °C. Injections consisted of 1 μl of 1 mM sample in DMSO over a gradient from 5% acetonitrile and 0.1% formic acid to 90% acetonitrile and 0.1% formic acid over a timespan of 2.5 min. Purity for each compound was determined from the integrated UV absorbance peak (Supplementary Fig. 16). Masses were detected in the positive ion scan and correspond to those predicted by their formula weight (Supplementary Fig. 16). The detector conditions were capillary voltage 3.25 kV, cone voltage 30 V, source temperature 150 °C, desolvation temperature 500 °C, desolvation gas 1,000 l h^−1^, cone gas 100 l h^−1^. Single ion recording was used to determine quantification of samples. The data were acquired over scan range from *m*/*z*=100–1,000 in 0.2 s and processed using QuanLynx software. Proton NMR spectra were acquired for each compound on a Bruker Ascend 400 MHz spectrometer to further assess the identity and purity of the samples. The indicated solvents correspond to those used in previous publications (pyridine for E7107 (ref. 9), chloroform for spliceostatin A^37^ and sudemycin D6 (ref. 38), and methanol for herboxidiene^39^) (Supplementary Fig. 16). The acquired spectra match previous data reported for these compounds.

### Resistant mutation identification by WXS

In total, 2.5 million HCT116 cells were seeded in each 10 cm dish and treated with indicated dosages of splicing modulators for 2 weeks. Compounds were refreshed every 4 days. When needed, confluent dishes were split 1:3 and cells were allowed to recover overnight without splicing modulator treatment after re-seeding. At the end of the compound selection period, surviving individual clones were picked and transferred to 12-well plates. Individual resistant clones were further expanded without splicing modulator treatment and one million cells from each clone were pelleted for genomic DNA extraction using the DNeasy Blood & Tissue Kit from Qiagen. WXS libraries were generated by Novogene Corporation using Agilent SureSelect Human All Exon V6 kit and sequenced on Illumina HiSeq platform. 12G raw data were gathered for each sample. WXS reads were then aligned to hg19 by BWA-MEM^40^ and somatic mutations were identified with MuTect2 (ref. 41) through Sentieon pipeline^42^ by pairing resistant clone with parental cell lines. As we selected the resistant clones for WXS, the allele frequencies for the mutations which are responsible for the resistance should be high. We focused on non-silent mutations (among the H3 curated spliceosome genes) with allele frequency higher than 0.2.

### Cell viability assay

For CellTiter-Glo analysis, 500 cells were seeded in each well of a 384-well plate the day before compound addition. An 11 pt serial dilution was used starting with a top final dosage of 10 μM for 10 additional doses. DMSO percentage was maintained throughout and a DMSO-only control was included. Seventy-two hours post compound addition, CellTiter-Glo reagent was added to the medium, incubated and assayed on EnVision Multilabel Reader (PerkinElmer). The luminescence value from each treatment sample was normalized to the average value of the respective DMSO control. The dosage response curve plots were generated using Graphpad Prism 6 and fit using nonlinear regression analysis and the log(inhibitor) versus response—Variable slope (four parameters). For heatmap summarization of IC_50_ shifts, IC_50_ value was extracted from dosage response curves and the fold changes of IC_50_ values in PHF5A variants expressing lines over that of the WT lines were calculated and plotted using TIBCO Spotfire software. For IC_50_s greater than the top dosage, the values were arbitrarily set at 10 μM. Unsupervised clustering analysis was performed in TIBCO Spotfire using the following default parameters: Clustering method: UPGMA; Distance measure: Euclidean; Ordering weight: Average value; Normalization: (None); Empty value replacement: Constant value: 0.

### Cell proliferation assay

One thousand cells of indicated genotypes were seeded in 96-well clear bottom plates (Corning, #3904) and HD phase-contrast image was captured every 4 h with × 4 objective lens using IncuCyte ZOOM System (Essen BioScience). Collected images were analysed with IncuCyte ZOOM Software (2016A) (Essen BioScience) to calculate the confluency percentage. Analysed data were graphed with Graphpad Prism 6, *n*=5.

### Immunofluorescence

One million cells of indicated genotypes were seeded onto Corning BioCoat Fibronectin 22 mm cover-slips (Fisher Scientific 08-774-386) in six-well plates. After 2 days, cells were fixed with 4% paraformaldehyde/phosphate-buffered saline (PBS) for 20 min at room temperature (RT). After 3 × PBS wash, cells were permeabilized with 0.1% Triton X-100/PBS for 20 min at RT. After 3 × PBS wash, cells were blocked with 5% FBS/PBS for 1 h at RT and incubated with α-SF3B1 mouse monoclonal antibody (MBL, D221-3) or α-SC35 mouse monoclonal antibody (Abcam, ab11826) at 1:50 dilution in 5% FBS/PBS in cold room overnight. On the second day, coverslips were washed with PBS three times and incubated with Alexa Fluor 488 anti-mouse secondary antibody (Thermo Fisher Cat #: A-11029) at 1:500 dilution in 5% FBS/PBS at RT in dark for 1 h. Coverslips were then washed with PBS three times and mounted using ProLong Gold Antifade Mountant with DAPI (Thermo Fisher, P36935). Slides were imaged with a × 40 objective on an Olympus IX-81 inverted fluorescence microscope and imaged, captured and processed with Metamorph for Olympus.

### Cell lysis and nuclear extract preparation

For western blot analysis, cell pellets were extracted using RIPA buffer supplemented with complete protease inhibitor cocktail and PhosStop phosphatase inhibitor cocktail (Roche Life Science). Lysates were then centrifuged for 10 min at top speed, and the supernatants were subjected to SDS–PAGE. For nuclear extract preparation, cells were first washed and then scraped into PBS. After centrifugation, cell pellets were resuspended in five packed cell volume of hypotonic buffer (10 mM HEPES, pH 7.9, 1.5 mM MgCl_2_, 10 mM KCl, 0.2 mM PMSF, 0.5 mM DTT) and centrifuged at 1,700*g* for 5 min. Cell pellets were resuspended in three packed cell volume of hypotonic buffer and swelled on ice for 10 min. Swollen cells were then lysed using a dounce homogenizer and spun at 1,500 g for 15 min at 4 °C. The pellets contained the nuclei and were suspended with ½ packed nuclei volume of low-salt buffer (20 mM HEPES, pH 7.9, 1.5 mM MgCl_2_, 20 mM KCl, 0.2 mM EDTA, 25% glycerol, 0.2 mM PMSF, 0.5 mM DTT) gently. ½ packed nuclei volume of high salt buffer (20 mM HEPES, pH 7.9, 1.5 mM MgCl_2_, 1.4 M KCl, 0.2 mM EDTA, 25% glycerol, 0.2 mM PMSF, 0.5 mM DTT) was then added and mixed gently. The lysates were rocked for 30 min in cold room before centrifuged at 9,000*g* for 30 min at 4 °C. The supernatants contained the nuclear extracts and were dialysed for 4 h using Slide-A-Lyzer dialysis cassettes with 30,000 MWCO cutoff in dialysis buffer (20 mM HEPES, pH 7.9, 0.2 mM EDTA, 20% glycerol, 0.2 mM PMSF, 0.5 mM DTT) with a change of buffer after 2 h. The nuclear extract was then aliquoted and flash frozen.

### *In vitro* splicing assay

The following Ad2-derived^43^ and subsequently modified^30^ sequence (actctcttccgcatcgctgtctgcgagggccagctgttggg*gtgagtactccctctcaaaagcgggcatgacttctgcgctaagattgtcagtttccaaaaacgaggaggatttgatattcacctggcccgcggtgatgcctttgagggtggccgcgtccatctggtcagaaaagacaatctttttgttgtcaagctttgcacgtctagggcgcagtagtccagggtttccttgatgatgtcatactaatcctgtcccttttttttccacag*ctcgcggttgaggacaaactcttcgcggtctttccagtactcttggatcggaaacccgtcggcctccgaacg) (intron in italics and underlined) was cloned into the pGEM-3Z vector (Promega) using 5′ *Eco*RI and 3′ *Xba*I restriction sites. The Ftzi plasmid was obtained from Robin Reed. The pGEM-3Z-Ad2.1 and Ftzi plasmids were linearized using *Xba*I and *Eco*RI, respectively, purified, resuspended in TE buffer and used as a DNA template in the *in vitro* transcription reaction. The Ad2.1 pre-mRNA and Ftz mRNA were generated and purified using MEGAScript T7 and MegaClear kits, respectively (Invitrogen). Twenty-microlitre splicing reactions were prepared using 80 μg nuclear extracts, 20 U RNAsin Ribonuclease inhibitor (Promega), 20 ng Ad2.1 pre-mRNA and 2 ng Ftz mRNA (internal control). After a 15-min pre-incubation with indicated compound, activation buffer (0.5 mM ATP, 20 mM creatine phosphate, 1.6 mM MgCl_2_) was added to initiate splicing, and the reactions were incubated for 90 min at 30 °C. RNA was extracted using a modified protocol from a RNeasy 96 Kit (Qiagen). The splicing reactions were quenched in 350 μl Buffer RLT Plus (Qiagen), and 1.5 volume ethanol was added. The mixture was transferred to an RNeasy 96 plate, and the samples were processed as described in the kit protocol. RNA was diluted 1 to 100 with dH_2_O. Ten microlitres RT–qPCR reactions were prepared using TaqMan RNA-to-C_T_ 1-step kit (Life Technologies), 2 μl diluted splicing reactions, 0.5 μl Ad2 mRNA primer/probe set and 0.5 μl Ftz mRNA primer/probe set. The Ad2 Ftz probes are from IDT and labelled with FAM acceptor with ZEN quencher and the Ftz probe is labelled with Hex and ZEN quencher, primer and probe set sequences are listed in Supplementary Table 1.

### Mass spectrometry analysis

The enriched samples were reduced with 5 mM DTT at 56 °C for 45 min and alkylated with 20 mM iodoacetamide at RT for 30 min. The samples were run on a 4–15% Tris glycine gel and the gel was excised, de-stained and trypsin digested overnight at 30 °C. Peptides were extracted with 50 μl of buffers A, B and C sequentially (Buffer A—1% formic acid and 50% acetonitrile, B—100 mM ammonium bicarbonate, C—100% acetonitrile). Samples were dried down using a lyophilizer and resuspended in 30 μl of running buffer A (0.1% formic acid in water). Samples were analysed by nanocapillary liquid chromatography tandem mass spectrometry on an easy-nLC 1,000 HPLC system coupled to a QExactive mass spectrometer (Thermo Scientific) using a C18 easy spray column particle size: 3 μm; 150 × 0.075 mm I.D. and the data were analysed using Proteome discoverer 1.4.

### Expression and crystallization of PHF5A

Full-length human PHF5A, containing a C40S mutation for enhanced protein stability, was synthesized and subcloned between the *Nde*I and *Eco*RI sites of pET-28a with an N-terminal His-MBP-TEV cleavable tag. The codon optimized sequence was: atggcaaaacaccatccggacttaatcttttgccgcaagcaggccggtgttgcaatcggccgtctgtgcgagaaatgcgacggcaagtgcgtgatctgtgacagctatgtgcgccctagtaccctggttcgcatctgcgacgagtgcaattatggcagctatcagggccgttgcgttatttgcggtggtccgggtgttagcgatgcctattactgcaaagaatgcaccattcaggaaaaggatcgcgatggctgtccgaagatcgttaacctgggcagcagcaaaaccgacctgttttacgaacgtaagaagtatggcttcaagaaacgctga. Protein was expressed in BL21 Star (DE3) cells (Thermofisher) grown in LB media. Cells were induced at OD_600_=1.0 overnight at 16 °C with 0.5 M IPTG supplemented with 100 μM ZnCl_2_. Lysate was prepared in HEPES pH 7.5, 500 mM NaCl, 1 mM TCEP, loaded onto a NTA-column and eluted over a gradient up to 500 mM imidazole. The peak fraction was pooled and the MBP tag was cleaved by TEV protease overnight at 4 °C. Cleaved MBP and excess TEV were removed by reverse NTA column. The flow through fractions containing PHF5A were concentrated and loaded onto a 16/60 Sephacryl-100 column equilibrated in 100 mM NaCl, 25 mM HEPES pH 7.5, 1 mM TCEP. The peak fraction was further purified by ion exchange on a HiTrap SP HP column equilibrated in gel filtration buffer and eluted in a gradient up to 1 M NaCl. PHF5A eluted in approximately 300 mM NaCl and was concentrated to 10 mg ml^−1^ and flash frozen in liquid N_2_ for storage at −80 °C. The resulting protein failed to crystallize but a proteolytically stable domain was obtained by limited digestion with chymotrypsin (1:1,000 molar ratio) for 2 h at RT. Cubic-shaped crystals grew to final dimensions of 50 × 50 × 50 μm after a week from 2 μl+2 μl hanging drops equilibrated over a reservoir containing 100 mM CHES pH 9.5, 800 mM sodium citrate and 0.5% octyl-β-glucoside. Crystals were frozen in reservoir solution supplemented with 20% ethylene glycol.

### Structure determination

Single wavelength anomalous diffraction (SAD) data at the zinc edge were collected by Shamrock Structures LLC at the APS beamline 21D (Table 1). Crystals diffracted to 2.0 Å and the data were processed with iMosflm and xia2 in a cubic space group P2_1_3 (*a*=*b*=*c*=82.2 Å and *α*=*β*=*γ*=90°)^44^,^45^ indicating a solvent content of 47%, assuming two molecules in the asymmetric unit. Anomalous signal extended to approximately 2.0 Å (Supplementary Fig. 17) and was used to locate six high-occupancy zinc anomalous sites using SHELX C/D/E^46^,^47^, confirming two molecules in the asymmetric unit. The FOM from this initial substructure solution was 0.404 and after density modification and hand determination, the FOM improved to 0.76. Buccaneer and REFMAC5 (ref. 48) auto-traced 76 residues for each monomer and we were able to model an additional 13 residues using Coot. This model was used to refine against the native data set to 1.8 Å and after several iterative rounds of rebuilding and refinement, the final model was obtained consisting of residues 2–91 in molecule A and 3–92 in molecule B and final statistics *R*=0.17, *R*_free_=0.20 and FOM=0.86 (Table 1)^48^,^49^. Representative electron density from the final 2Fo–Fc map, contoured at 1*σ*, is shown (Supplementary Fig. 18).

### Cloning and purification of the recombinant protein complex

To reassemble the modulator-binding site, four proteins from the SF3b complex were selected based on the yeast cryo-EM structure^15^. Truncated SF3B1, full-length SF3B3, PHF5A and SF3B5 were synthesized and subcloned between the *Eco*RI and *Nco*I site of pFastBac1 vector. Only the HEAT repeat domain from residue 454–1,304 of SF3B1 was cloned with an addition of N-terminal FLAG tag. SF3B3 and SF3B5 were with an N-terminal His-tag. Four viruses were generated and used to co-infect SF21 cells at a ratio of ∼10:1. The cells were harvested after 72 h and lysed in 40 mM HEPES pH 8.0, 500 mM NaCl, 10% glycerol and 1 mM TCEP. The complex was purified by batch method, using nickel beads and FLAG beads. The eluent was concentrated and ran on a gel filtration column (superdex 200) in buffer 20 mM HEPES pH 8.0, 300 mM NaCl, 10% glycerol and 1 mM TCEP. The fraction was collected, concentrated to 4 mg ml^−1^ and flash frozen in liquid N_2_ for storage at −80. The production of recombinant complex containing PHF5A-Y36C mutation is the same as the WT recombinant complex.

### Scintillation proximity assay

Batch immobilization of anti-FLAG antibody (Sigma) to anti-mouse PVT SPA scintillation beads (PerkinElmer) was prepared as follows. For every 1.5 mg of beads, 10 μg antibody was prepared in 150 μl PBS. The antibody–bead mixture was incubated for 30 min at RT and centrifuged at 18,000g for 5 min. One hundred and fifty microlitres PBS was used to resuspend every 1.5 mg antibody–bead mixture. The aforementioned mini-SF3b complexes were tested for ^3^H-labelled pladienolide probe^9^ binding. One hundred microlitres binding reactions were prepared with 50 μl bead slurry and 0 or 50 nM protein in buffer (20 mM HEPES pH 8, 200 mM KCl, 5% glycerol). The mixture was incubated for 30 min, and varying concentrations of ^3^H-labelled pladienolide probe were added. The mixture was incubated for 30 min, and luminescence signals were read using a MicroBeta2 Plate Counter (PerkinElmer). Compound competition studies were performed with the WT mini-SF3b complex. One hundred microlitres binding reactions were prepared with 50 μl bead slurry, 25 nM protein in buffer and compounds at varying concentrations. After a 30-min pre-incubation, 1 nM ^3^H-labelled pladienolide probe was added. The reactions were incubated for 30 min, and luminescence signals were read.

Previously prepared nuclear extracts were stored as 2.5 mg aliquots. Each aliquot was sufficient for three SPA samples and was diluted into a total volume of 1 ml PBS with phosphatase and protease inhibitors. Sufficient amounts of aliquots were centrifuged at 18,000*g* for 10 min at 4 °C. The supernatant was removed into a clean tube and kept on ice. Batch immobilization of anti-SF3B1 antibody (MBL) to anti-mouse PVT SPA scintillation beads (PerkinElmer) was prepared as follows. For every 2.5 mg of nuclear extracts, 5 μg anti-SF3B1 antibody and 1.5 mg of beads were mixed in 150 μl PBS. The antibody–bead mixture was incubated for 30 min at RT and centrifuged at 18,000*g* for 5 min. The beads were suspended and added to the prepared nuclear extracts. The slurry was incubated for 2 h at 4 °C with gentle mixing. The beads were collected by centrifuging at 18,000*g* for 5 min, and washed twice with PBS+0.1% Triton X-100. After a final centrifugation step, every 1.5 mg of beads was suspended with 150 μl of PBS. One hundred microlitres binding reactions were prepared as follows: 50 μl bead slurry, 25 μl cold competitive compound at 10 μM, and after 30 min pre-incubation, 10 nM ^3^H-labelled pladienolide probe was added. The mixture was incubated for 30 min, and luminescence signals were read using a MicroBeta2 Plate Counter (PerkinElmer).

### RNA-Seq and data analysis

Either PHF5A WT or Y36C mutant-overexpressing cells were treated with either DMSO or E7107 (100 nM and 10 μM) for 6 h in hexaplicate before lysed in TRIzol reagent (Thermo Fisher). After phase separation, the top aqueous phase was further processed using MagMAX-96 Total RNA Isolation Kit (Thermo Fisher, AM1830) for RNA extraction. RNA quality was assessed using Agilent tapestation with RNA screen tape. RNA-seq libraries were prepared by Beijing Genomic Institute (BGI) and sequenced on Illumina Hiseq 4,000 for 6G clean reads per sample. RNA-seq reads were aligned to hg19 by STAR^50^ and raw junction counts generated by STAR were used for calculating PSI to quantify splice junction usage relative to all other splice junctions that share the same splice site as described before^51^. Differential PSI were assessed between a pair of sample groups using moderated *t*-test defined in limma package^52^ in Bioconductor. The statistical *P*-values were corrected using the Benjamini–Hochberg procedure and *q*-values ≤0.05 were considered statistically significant. Gene IDs associated with significant splicing changes upon E7107 treatment as compared to DMSO in either PHF5A WT or Y36C cells were used for generation of the Venn Diagram using online tool (http://bioinformatics.psb.ugent.be/webtools/Venn/). PHF5A WT- or Y36C-specific genes identified from the Venn Diagram analysis were then subject to Gene Set Enrichment Analysis (http://software.broadinstitute.org/gsea/msigdb/annotate.jsp) using the Kyoto Encyclopedia of Genes and Genomes (KEGG) pathway database.

### ES versus IR PSI comparison

The number of reads which cover the splice junction which excludes a given cassette exon (ES reads) are compared with both the number of spliced reads which share its 3′ splice site yet have an alternative 5′ splice site bordering the cassette exon (exon inclusion reads) and the number of reads which cross the exon–intron boundary at that same 3′ splice site (IR reads). These counts are summed and their fractions from the PSI for the ES event, the exon inclusion event and the IR event, respectively, at that locus. The PSI for all significant ES events derived from the comparison between 100 nM E7107 treatment in PHF5A Y36C cells and the respective DMSO controls (3,883 events) and the PSI for the IR junction at the same locus are plotted in blue and green, respectively. For all other treatments, the PSI of the ES junction and the IR junction for each locus is plotted in the same order. PSI is averaged over samples in hexaplicate.

### GC content calculation of retained intron junctions

The set of all significantly retained intron junctions was reduced to those which had an intron of length at least 100 and which bordered at least one exon of length 50 from RefSeq at its 3′ end. If multiple exons of length 50 were found, one was randomly selected. The sequences of each intron and exon were divided into 100 and 50 bins of equal length strings, respectively, then the GC content (fraction of bases either ‘G' or ‘C') was assessed for each string. Once all intron/exon pairs have their sequence content binned in this way, the resulting mean and 95% confidence interval for each bin were assessed using 100 bootstraps of the data (up to the number of intron/exon pairs, with replacement) and drawn using a solid line and a transparent interval, respectively. The background, in grey, was drawn from 10,000 random intron/exon pairs from RefSeq which satisfied the same length and boundary requirements.

### GC content calculation of ES junctions

The set of all significant, treatment-induced ES junctions was reduced to those for which both introns (those bordering the cassette exon on their 3′ and 5′ ends, respectively) had a sequence length of at least 100, were significantly enriched in the untreated samples as ‘exon inclusion' events with *q*<0.05, and for which the intervening sequence space formed by the borders of their 3′ and 5′ ends was known to be an exon in the RefSeq transcriptome annotation of length at least 50, to avoid ambiguity caused by events which skip multiple exons. The sequences of each intron and exon were divided into 100 and 50 bins of equal length strings, respectively, then the GC content (fraction of bases either ‘G' or ‘C') were assessed for each string. Once all intron/exon pairs have their sequence content binned in this way, the resulting mean and 95% confidence interval for each bin were assessed using 100 bootstraps of the data (up to the number of intron/exon pairs, with replacement) and drawn using a solid line and a transparent interval, respectively. The background, in grey, was drawn from 10,000 random intron/exon pairs from RefSeq, which satisfied the same length and boundary requirements.

### Taqman gene expression assay

Eight thousand cells of indicated genotypes were seeded in each well of 96-well plate and allowed to settle overnight. On the second day, 11 pt serial dilution (1:4 fold dilution across) of indicated compound with a top dosage of 10 μM final was added to the culturing medium. Four hours post compound addition, culturing medium was decanted and washed once with PBS. PBS was then decanted completely from the plate and Lysis buffer (plus DNase I) from TaqMan Gene Expression Cells-to-CT Kit (Thermo Fisher, cat # AM1729) was added according to the manual. After 5 min incubation at RT on the shaker, stop solution was added to each well and incubated for 2 min. Reverse transcription was set up immediately using the Cells-to-CT Kit and cDNAs were used for quantitative real-time PCR analysis using Viia7 (Thermo Fisher). Each reaction is multiplexed with an FAM-labelled probe targeting specific target gene splicing isoforms and a VIC-labelled probe targeted 18S rRNA as loading control. Therefore, the FAM Ct value in each well was first normalized to the VIC Ct value in the same well before further normalization to the FAM/VIC ratio of DMSO-treated control samples to calculate fold change over DMSO. Graphs were generated using Graphpad Prism 6, *n*=2. Taqman gene expression probes used in these assays are listed in Supplementary Table 1.

### Statistics

Appropriate statistical methods and determination of statistical significance were performed as described in specific Method sections.

### Data availability

Coordinates and structure factors were deposited in the Protein Data Bank (PDB accession code: 5SYB). UniProt entries Q7RTV0 and PDB accession codes 5SYB, 5GM6 and 2K0A were used in this study. RNA-seq data have been deposited in NCBI's Gene Expression Omnibus (GEO) repository and are accessible through GEO Series accession number GSE96917 (https://www.ncbi.nlm.nih.gov/geo/query/acc.cgi?acc=GSE96917). All other data are available from the corresponding authors upon reasonable request.

## Additional information

**How to cite this article:** Teng, T. *et al*. Splicing modulators act at the branch point adenosine binding pocket defined by the PHF5A–SF3b complex. *Nat. Commun.* **8,** 15522 doi: 10.1038/ncomms15522 (2017).

**Publisher's note**: Springer Nature remains neutral with regard to jurisdictional claims in published maps and institutional affiliations.

## Supplementary Material

Supplementary Information Supplementary Figures, Supplementary Tables.

Supplementary Data 1 Recurring Mutations Identified in Splicing Modulator Resistant Clones.

Supplementary Data 2 Curated Splicing Related Gene List.

Supplementary Data 3 Differential Splicing Analysis Junction Summary.

Supplementary Data 4 Metadata for 2470 splicing junctions that show decreased Exon Skipping and increased Intron Retention in E7107 treated PHF5A WT cell lines compared to E7107 treated PHF5A Y36C cell lines.

Supplementary Data 5 Gene Set Enrichment analysis of splicing modulator regulated genes in PHF5A WT and PHF5A Y36C cell lines

Supplementary Data 6 List of Genes with significant Intron Retention events in E7107 treated PHF5A WT cell lines and significant Exon Skipping events in E7107 treated PHF5A Y36C cell lines.

## Figures and Tables

**Table 1:** X-ray data collection and refinement statistics.

	**Peak (Zn edge)**	**Native (PDB: 5SYB)**
*Data collection*		
Space group	P2_1_3	P2_1_3
Cell dimension		
*a*, *b*, *c* (Å)	82.1	82.1
*α*, *β*, *γ* (°)	90.0	90.0
Wavelength (Å)	1.2781	1.0000
Resolution (Å)	33.53–2.04 (2.09–2.04)	58.06–1.80 (1.86–1.80)
*R*_means_ (%)	10.4 (78.8)	14.8 (>100%)
*I*/*σ*(*I*)	34.8 (6.3)	15.2 (2.7)
Completeness (%)	100 (98.3)	100 (100)
Redundancy	38.9 (38.1)	10.0 (8.1)
		
*Refinement*		
Resolution (Å)	33.53–2.04 (2.09–2.04)	58.06–1.80 (1.86–1.80)
No. reflections	469,101 (33,425)	318,806 (23,506)
*R*_work_/*R*_free_ (%)		17/20
No. of atoms		
Protein		1,483
Water		97
Ion		6
B factors		
Protein		33.9
Water		38.3
RMS deviations		
Bond lengths (Å)		0.02
Bond angles (°)		1.98
