# Supplementary material for: Splicing modulators act at the branch point adenosine binding pocket defined by the PHF5A–SF3b complex
Source: Nat Commun. 2017 May 25;8:15522. doi: 10.1038/ncomms15522 (PMC5458519; doi:10.1038/ncomms15522)
Supplement: Supplementary Information — Supplementary Figures, Supplementary Tables. [file ncomms15522-s1.pdf]

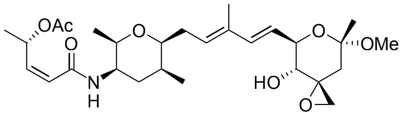

Spliceostatin A

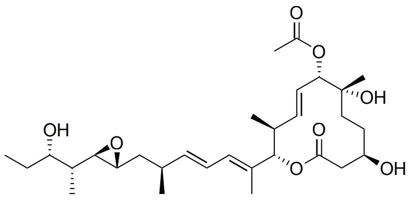

Pladienolide B

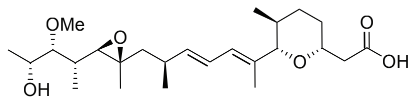

Herboxidiene

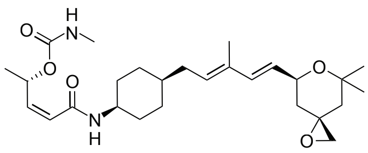

Sudemycin D6

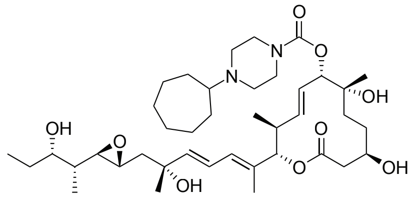

E7107

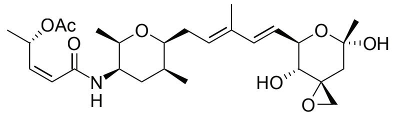

FR901464

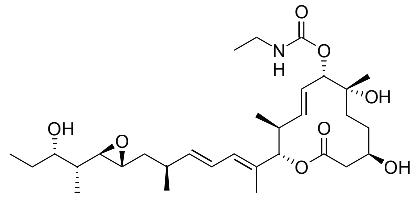

^{3}H labelled Pladienolide derivative

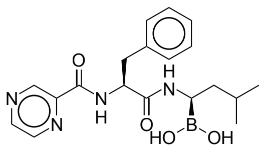

Bortezomib

Supplementary Figure 1: Structure of splicing-modulating small molecule compounds and a proteasome inhibitor bortezomib.

4nM E7107 resistant clone#1
SF3B1 residue 1078
WT = GTC = Valine
Mut = GCC = Alanine

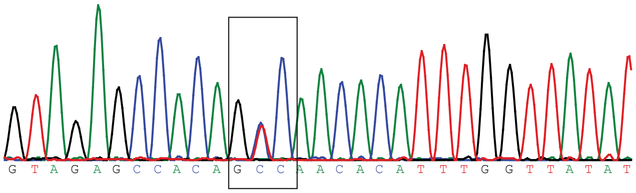

4nM E7107 resistant clone#3
PHF5A residue 36
WT = TAT= Tyrosine
Mut = TGT = Cysteine

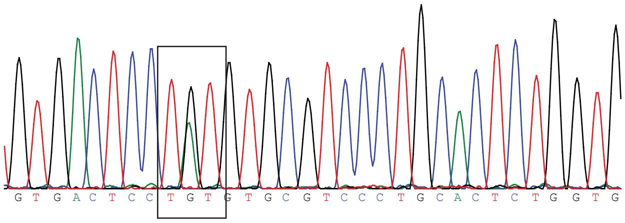

4nM E7107 resistant clone#4
SF3B1 residue 1074
WT = CGT = Arginine
Mut = CAT = Histidine

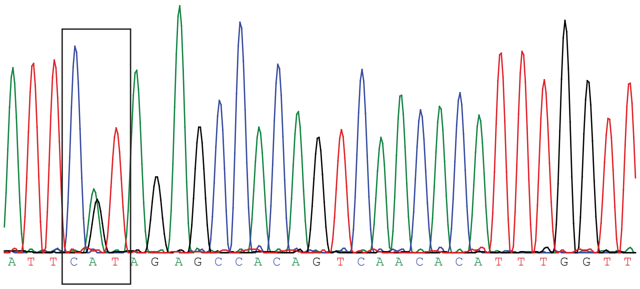

4nM E7107 resistant clone#6
SF3B1 residue 1078
WT = GTC = Valine
Mut = ATC = Isoleucine

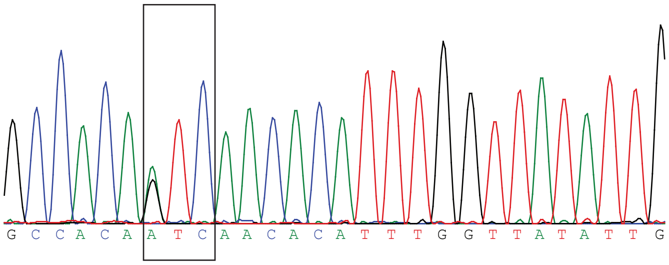

4nM E7107 resistant clone#7
SF3B1 residue 1078
WT = GTC = Valine
Mut = GCC = Alanine

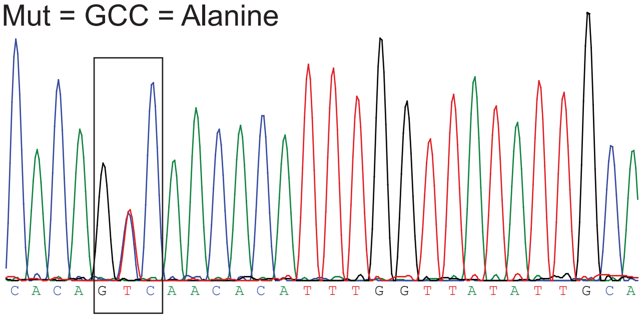

4nM E7107 resistant clone#8
SF3B1 residue 1078
WT = GTC = Valine
Mut = ATC = Isoleucine

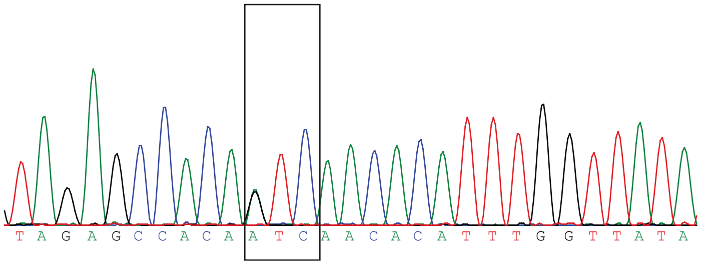

20nM Herboxidiene resistant clone#1
PHF5A residue 36
WT = TAT= Tyrosine
Mut = TGT = Cysteine

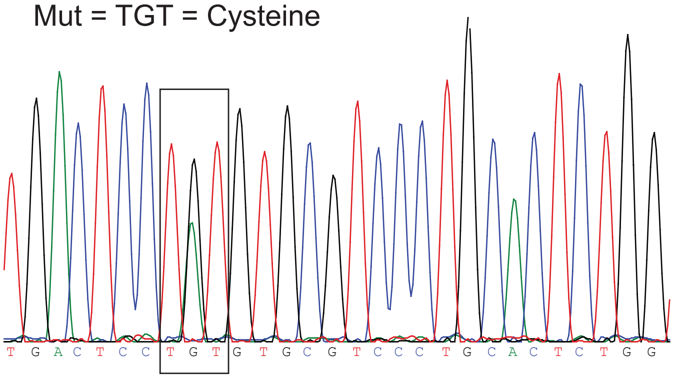

20nM Herboxidiene resistant clone#2
PHF5A residue 36
WT = TAT= Tyrosine
Mut = TGT = Cysteine

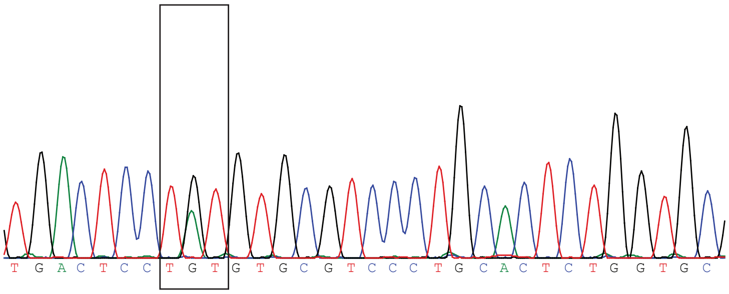

Supplementary Figure 2: Targeted Sanger sequencing of resistant clones.

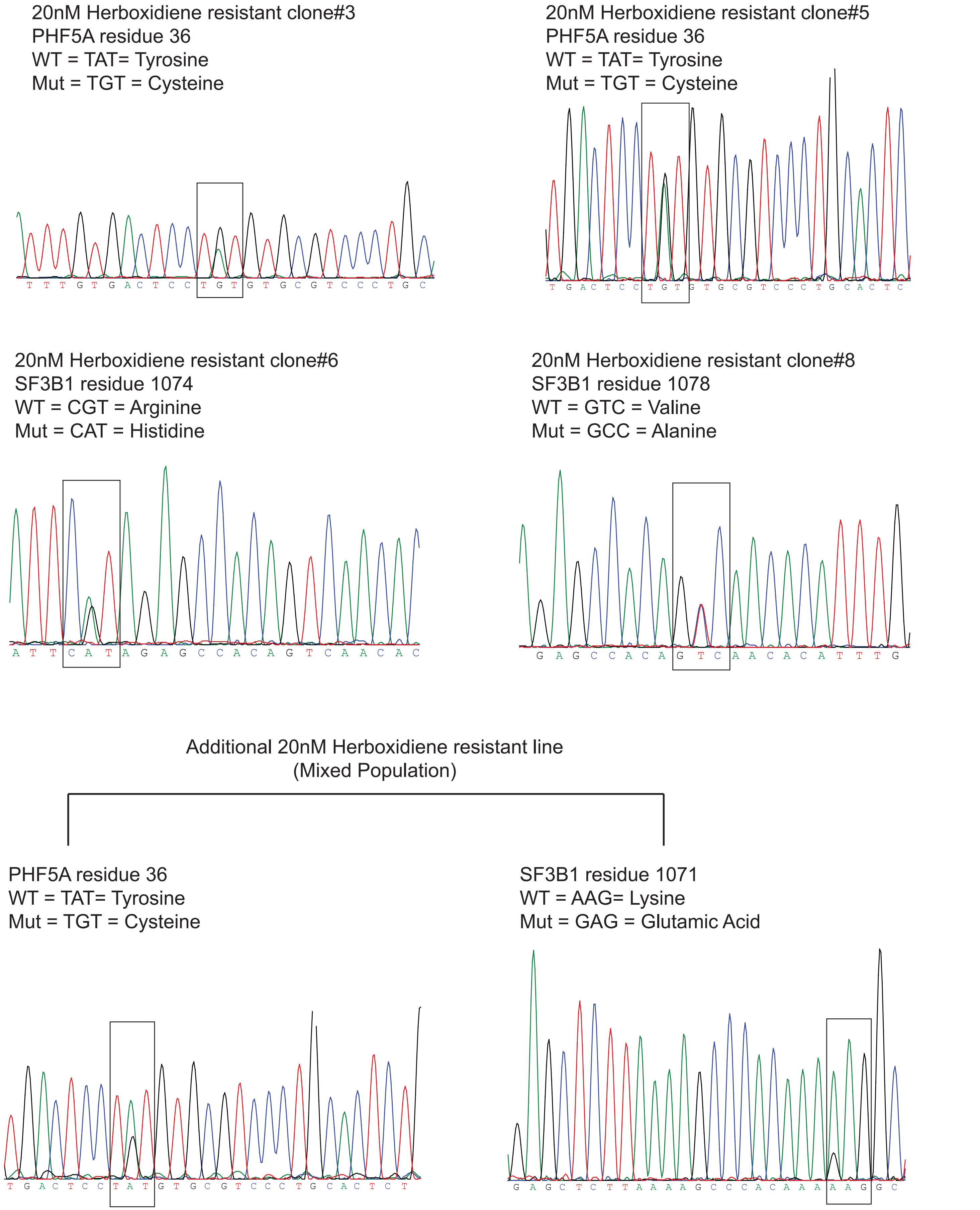

Supplementary Figure 2: Targeted Sanger sequencing of resistant clones (continued). Black box indicates affected codons, the respective coding and amino acid change were shown above sequencing results.

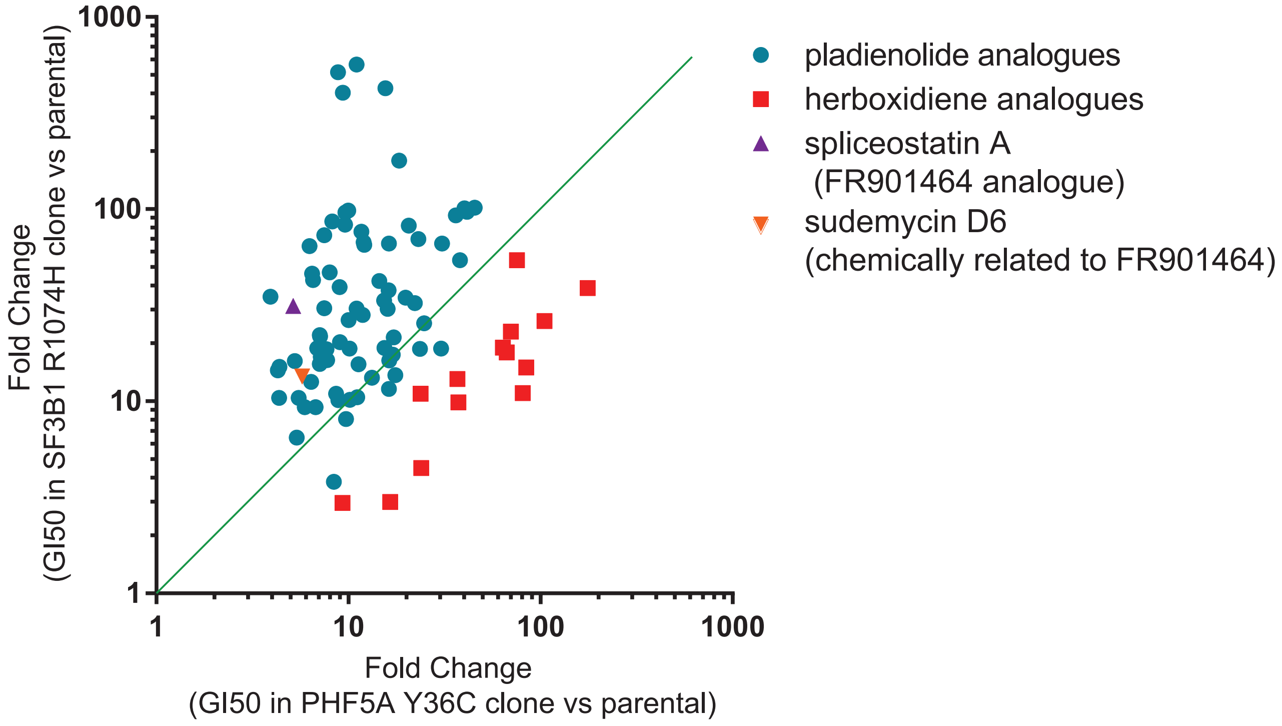

Supplementary Figure 3: PHF5A Y36C presents better resistance to herboxidiene analogues than pladienolide and FR901464 analogues whereas SF3B1 R1074H mutation presents with the opposite preference. X-axis is the GI50 ratios between the PHF5A Y36C mutation carrying clone versus the parental line of the same compound in logarithm scale. Y-axis is the GI50 ratios between the SF3B1 R1074H mutation carrying clone versus the parental line in logarithum scale. Green line is at 45° diagonal representing equal GI50 shift of the same compound in both resistant clones as compared to the parental line.

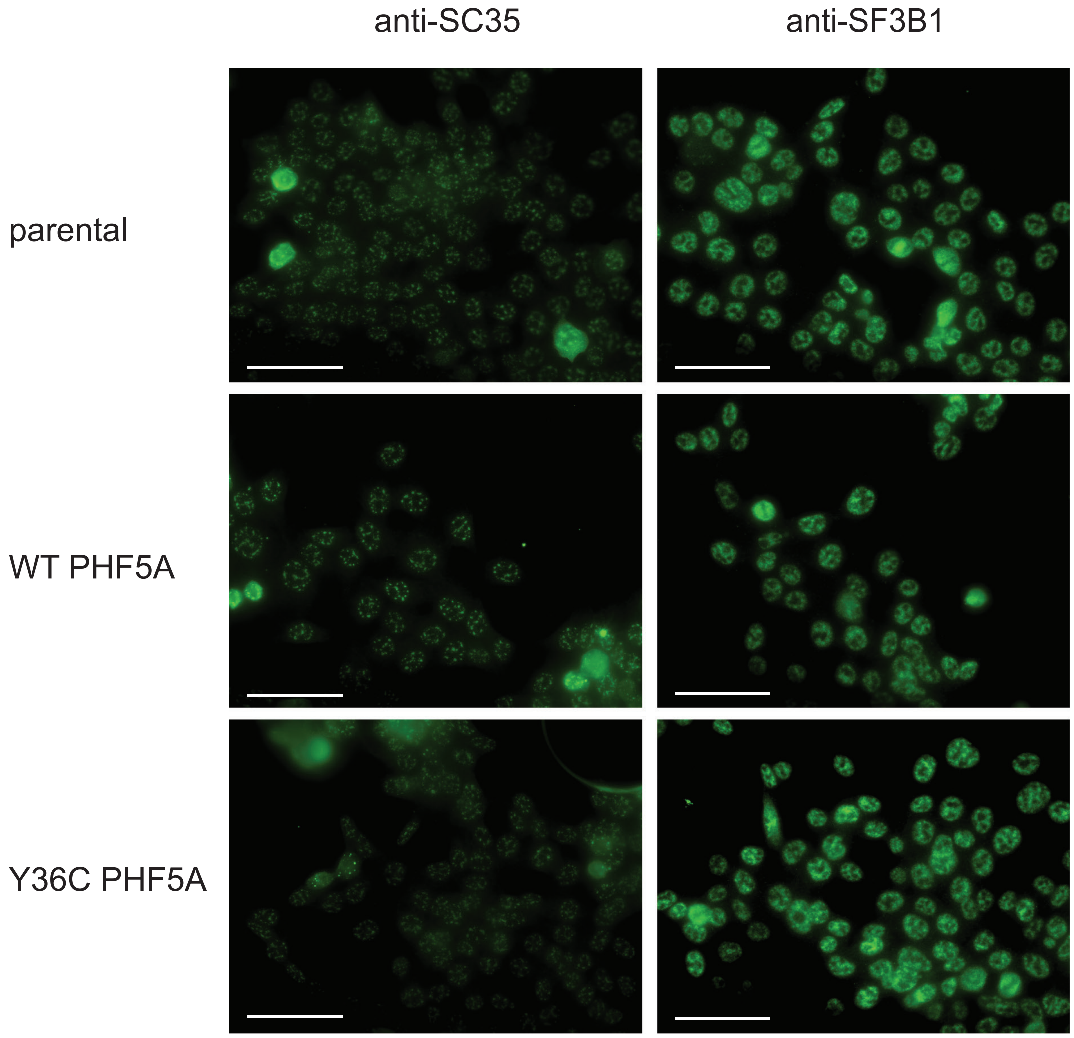

Supplementary Figure 4: Expression of PHF5A Y36C does not impact nuclear speckles formation or SF3B1 localization. Nuclear speckles were immuno-stained with anti-SC35 antibody. All scale bars indicate 50μM length.

Replicate 1

Wild Type – PHF5A

Description	Coverage(%)	# Peptides	MW [kDa]
SF3B1	61	73	145.7
SF3B2	52	46	100.2
SF3B3	58	58	135.5
SF3B5	46	2	10.1
SF3B6/P14	54	8	14.6
PHF5A	22	3	12.4

Mutant (Y36C) – PHF5A

Description	Coverage(%)	# Peptides	MW [kDa]
SF3B1	60	62	145.7
SF3B2	40	34	100.2
SF3B3	47	40	135.5
SF3B5	70	4	10.1
SF3B6/P14	43	6	14.6
PHF5A	30	4	12.4

Replicate 2

Wild Type – PHF5A

Description	Coverage(%)	# Peptides	MW [kDa]
SF3B1	62	70	145.7
SF3B2	48	39	100.2
SF3B3	54	53	135.5
SF3B6/P14	50	5	14.6
PHF5A	40	3	12.4

Mutant (Y36C) – PHF5A

Description	Coverage(%)	# Peptides	MW [kDa]
SF3B1	63	67	145.7
SF3B2	53	42	100.2
SF3B3	57	56	135.5
SF3B6/P14	50	5	14.6
PHF5A	20	2	12.4

Supplementary Figure 5: PHF5A Y36C does not change the composition of SF3b complex. Mass spectrometry analysis of SF3b complex proteins from SF3B1 pull-down from respective nuclear extract lysates, replicate 1 come from the same samples used for Fig. 2c and parallel samples from Supplementary Fig 13.

a

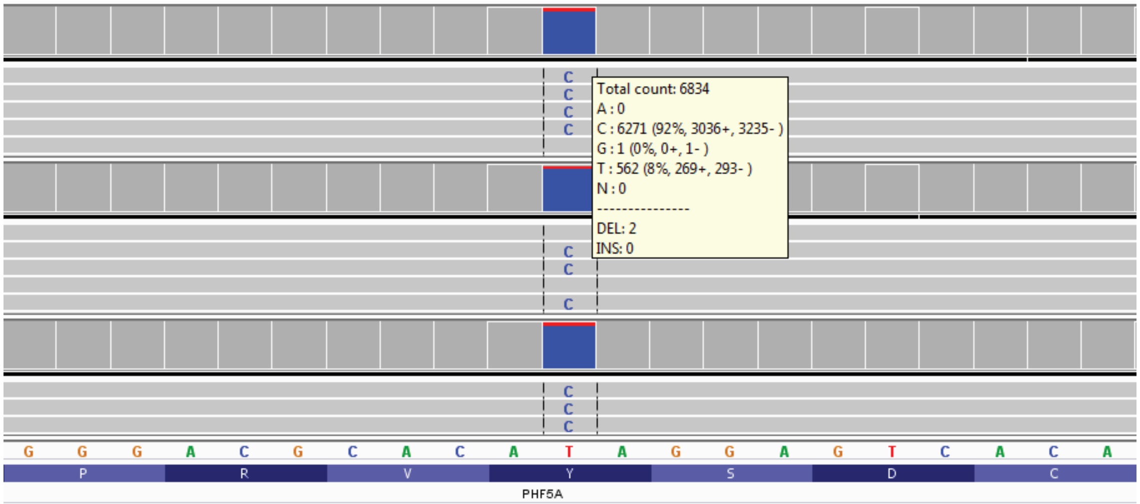

b

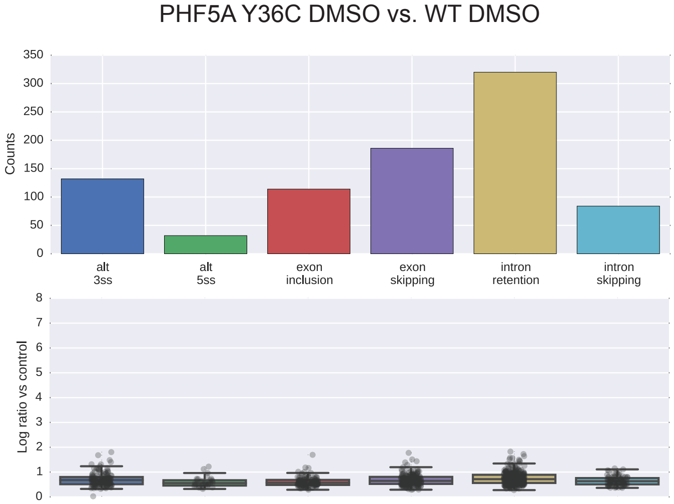

Supplementary Figure 6: PHF5A Y36C does not significantly impact global splicing. (a) Y36C PHF5A accounts for ~92% of total PHF5A mRNA expression in the engineered PHF5A Y36C over-expressing lines. Screenshot from IGV browser is shown. Note the sequence below is of reverse complimentary and the T to C conversion is corresponding to tyrosine to cycsteine change. (b) QC summary of differential splicing analysis between PHF5A Y36C cells treated with DMSO versus that of PHF5A WT cells showed minimal difference between two genotypes as measured by number of events and average fold change. Box shows the inter-quartile range (IQR) of the dataset whereas the whiskers illustrate 1.5xIQR. QC summary of parallel splicing modulator treated samples can be found in Fig. 4 for comparison.

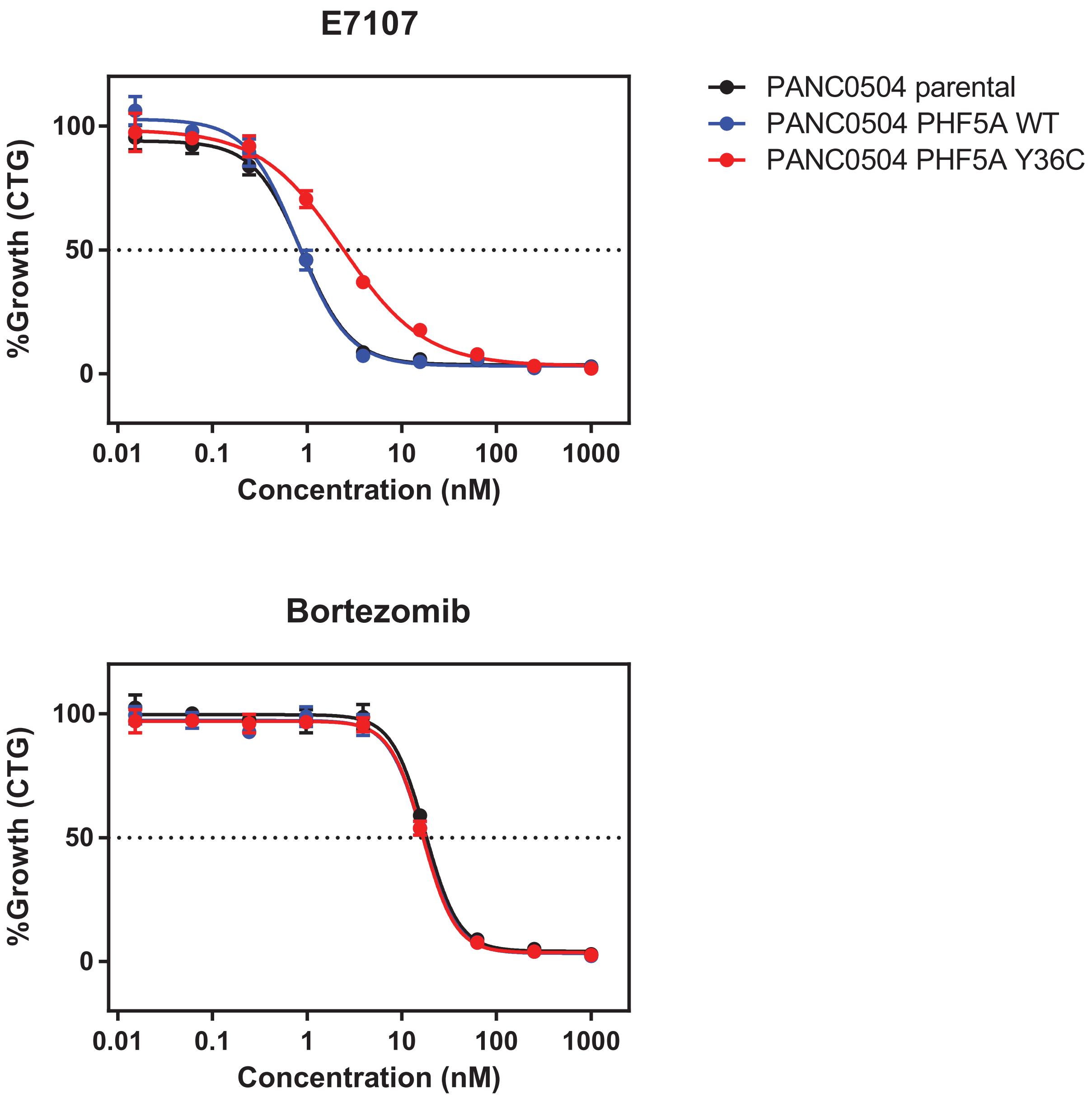

Supplementary Figure 7: PHF5A Y36C over-expression in PANC0504 cells shows partial resistant phenotype to splicing modulator E7107 but not proteasome inhibitor Bortezomib. Error bar indicates standard deviation, n=3.

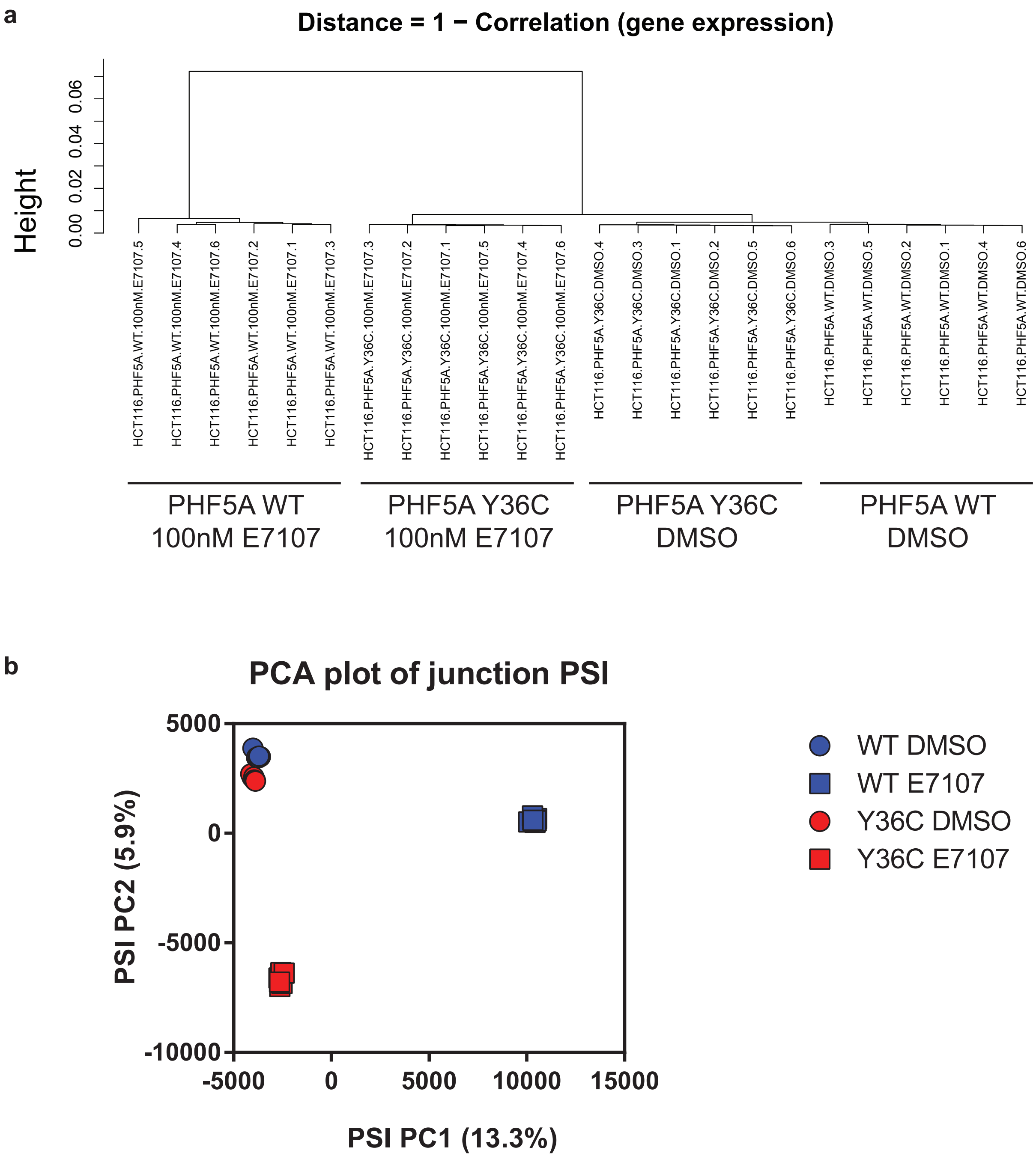

Supplementary Figure 8: QC summary of whole transcriptome RNA-seq analyses. (a) Unsupervised clustering of gene expression from the RNA-seq samples. (b) Principle component plot of splicing junction PSI (percent spliced in) from the RNA-seq samples.

a

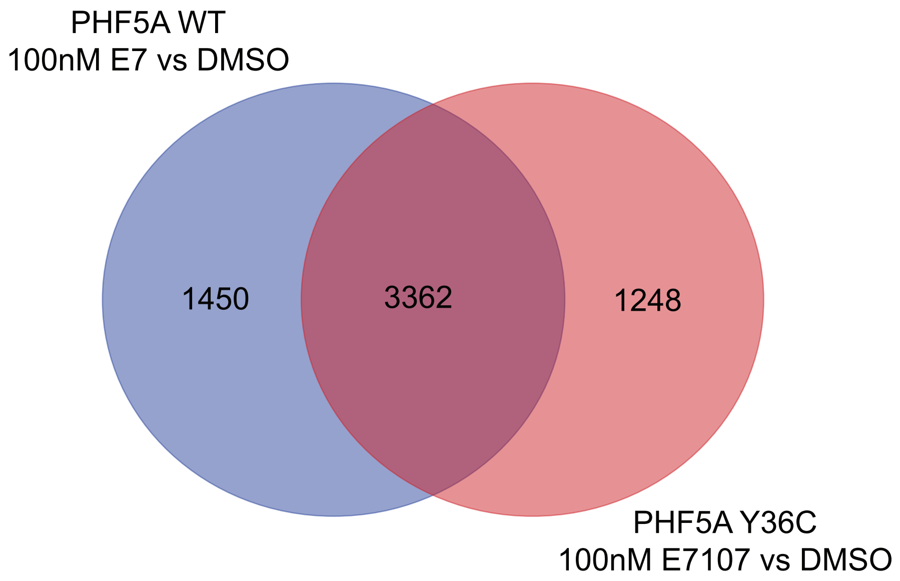

b PHF5A WT (100nM E7 vs DMSO) specific

Gene Set Name	# Genes in Gene Set (K)	# Genes in Overlap (k)	k/K	p-value	FDR q-value
KEGG_RIBOSOME	88	23	0.2614	7.23E-16	1.35E-13
KEGG_HUNTINGTONS_DISEASE	185	25	0.1351	2.71E-10	1.82E-08
KEGG_PATHWAYS_IN_CANCER	328	34	0.1037	2.93E-10	1.82E-08
KEGG_MAPK_SIGNALING_PATHWAY	267	30	0.1124	4.69E-10	2.18E-08
KEGG_SPLICEOSOME	128	20	0.1562	1.24E-09	4.60E-08
KEGG_RNA_POLYMERASE	29	10	0.3448	5.73E-09	1.78E-07
KEGG_CHRONIC_MYELOID_LEUKEMIA	73	14	0.1918	2.54E-08	5.98E-07
KEGG_BLADDER_CANCER	42	11	0.2619	2.58E-08	5.98E-07
KEGG_PYRIMIDINE_METABOLISM	98	16	0.1633	2.89E-08	5.98E-07
KEGG_ALZHEIMERS_DISEASE	169	20	0.1183	1.52E-07	2.83E-06

c PHF5A Y36C (100nM E7 vs DMSO) specific

Gene Set Name	# Genes in Gene Set (K)	# Genes in Overlap (k)	k/K	p-value	FDR q-value
KEGG_RENAL_CELL_CARCINOMA	70	14	0.2	3.46E-09	3.35E-07
KEGG_REGULATION_OF_ACTIN_CYTOSKELETON	216	24	0.1111	3.60E-09	3.35E-07
KEGG_MAPK_SIGNALING_PATHWAY	267	26	0.0974	1.33E-08	8.22E-07
KEGG_ALZHEIMERS_DISEASE	169	20	0.1183	2.45E-08	9.88E-07
KEGG_UBIQUITIN_MEDIATED_PROTEOLYSIS	138	18	0.1304	2.66E-08	9.88E-07
KEGG_PURINE_METABOLISM	159	19	0.1195	4.64E-08	1.44E-06
KEGG_HUNTINGTONS_DISEASE	185	20	0.1081	1.12E-07	2.96E-06
KEGG_LYSOSOME	121	16	0.1322	1.28E-07	2.99E-06
KEGG_PYRIMIDINE_METABOLISM	98	14	0.1429	2.95E-07	6.10E-06
KEGG_PARKINSONS_DISEASE	133	16	0.1203	4.79E-07	8.91E-06

Supplementary Figure 9: Gene level analysis of splicing modulator regulated genes. (a) Venn Diagram showing the overlap of genes regulated by 100nM E7107 as compared to DMSO in PHF5A WT cells (purple) and genes regulated by 100nM E7107 in Y36C (pink) cells. (b) Gene Set Enrichment Analysis (GSEA) using the Kyoto Encyclopedia of Genes and Genomes (KEGG) pathway database of PHF5A WT specific genes identified from (a). (c) GSEA using KEGG pathway database of PHF5A Y36C specific genes identified from (a). Only the top ten enriched pathways were shown in (b) and (c). Raw data used for generation of (a)(b)(c) were included as supplementary Table 6.

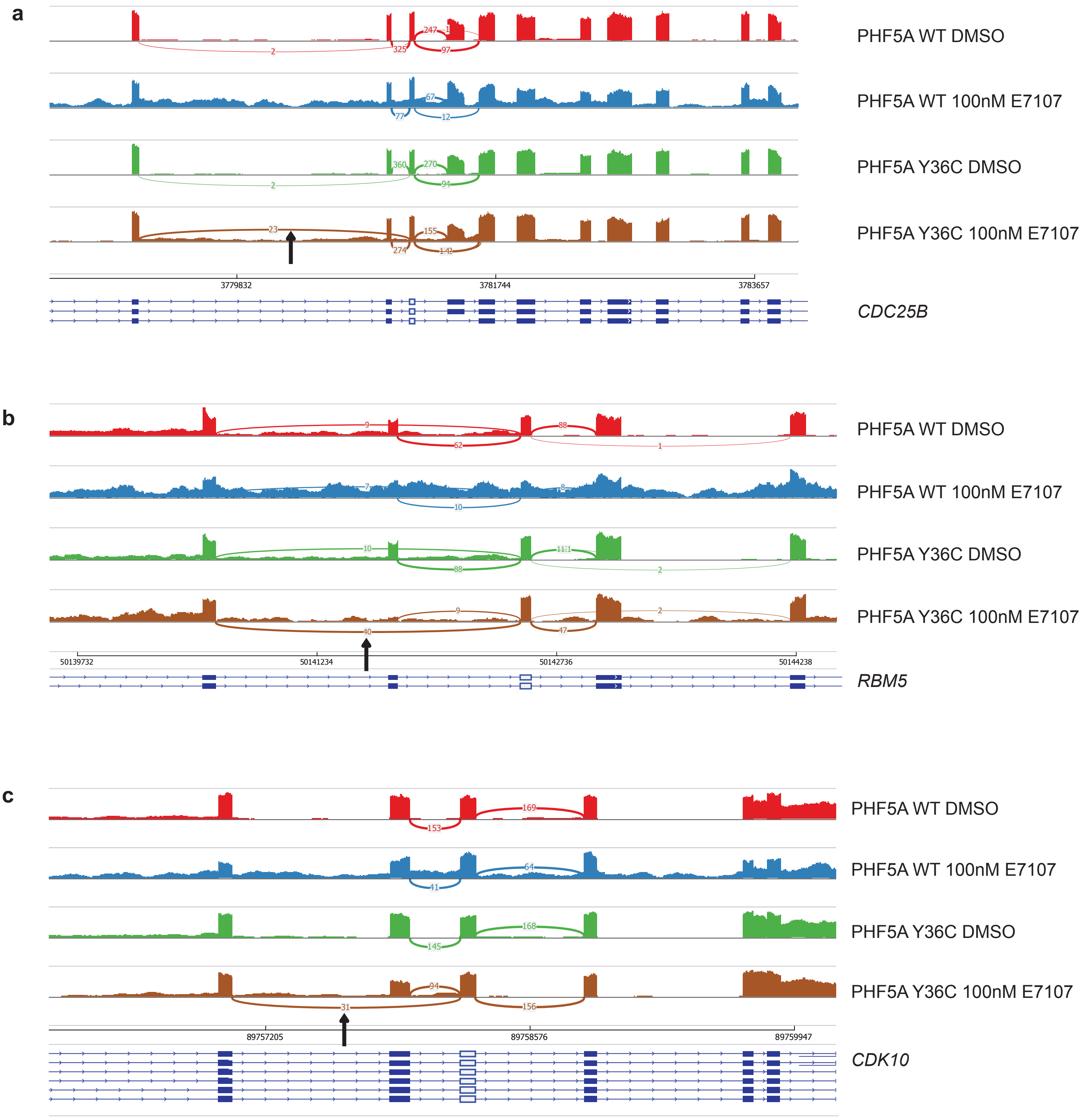

Supplementary Figure 10: Sashimi plots of representative gene junctions showing a intron-retension to exon-skipping event switch under E7107 treatment in PHF5A Y36C cells as compared to PHF5A WT cells. (a) *CDC25B*, (b) *RBM5*, (c) *CDK10*, arrow indicate the affected junction, only RNAseq reads splitting between affected junctions were shown.

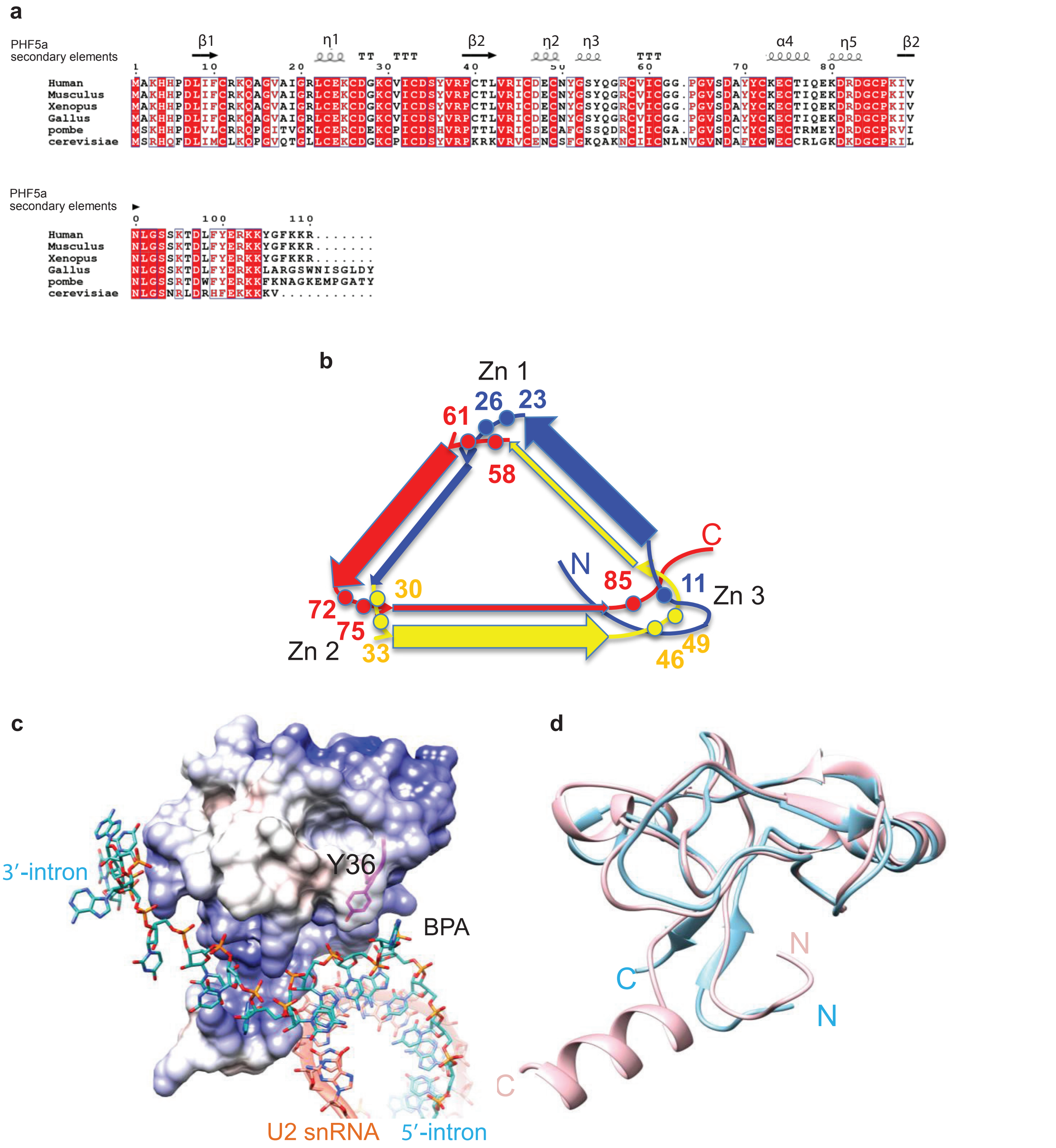

Supplementary Figure 11: (a) Sequence alignment of PHF5A. Strict identity are in white on a red background and similarity score higher than 0.2 are colored in red and framed in blue. The rest are weakly similar. (α: helix, β: strands, η:310 helix, T: turn) (b) Topology diagram representing a simple view of the triangular shape of PHF5A. An arrow represents 10 residues composing a side of triangles. Large arrows are the peptides closer to viewers and small arrows are away from viewers. Every 20 residues make two arrows and are colored in blue, yellow, red sequentially. Each vertex has a zinc ion coordinated with four cysteines and cysteines are shown as balls. (c) Surface view PHF5A model (Y36 in pink sticks) zoomed in to the interface of Rds3 and intron RNA (cyan sticks). (d) Superimposition of PHF5A (light blue) and Rds3 (pink).

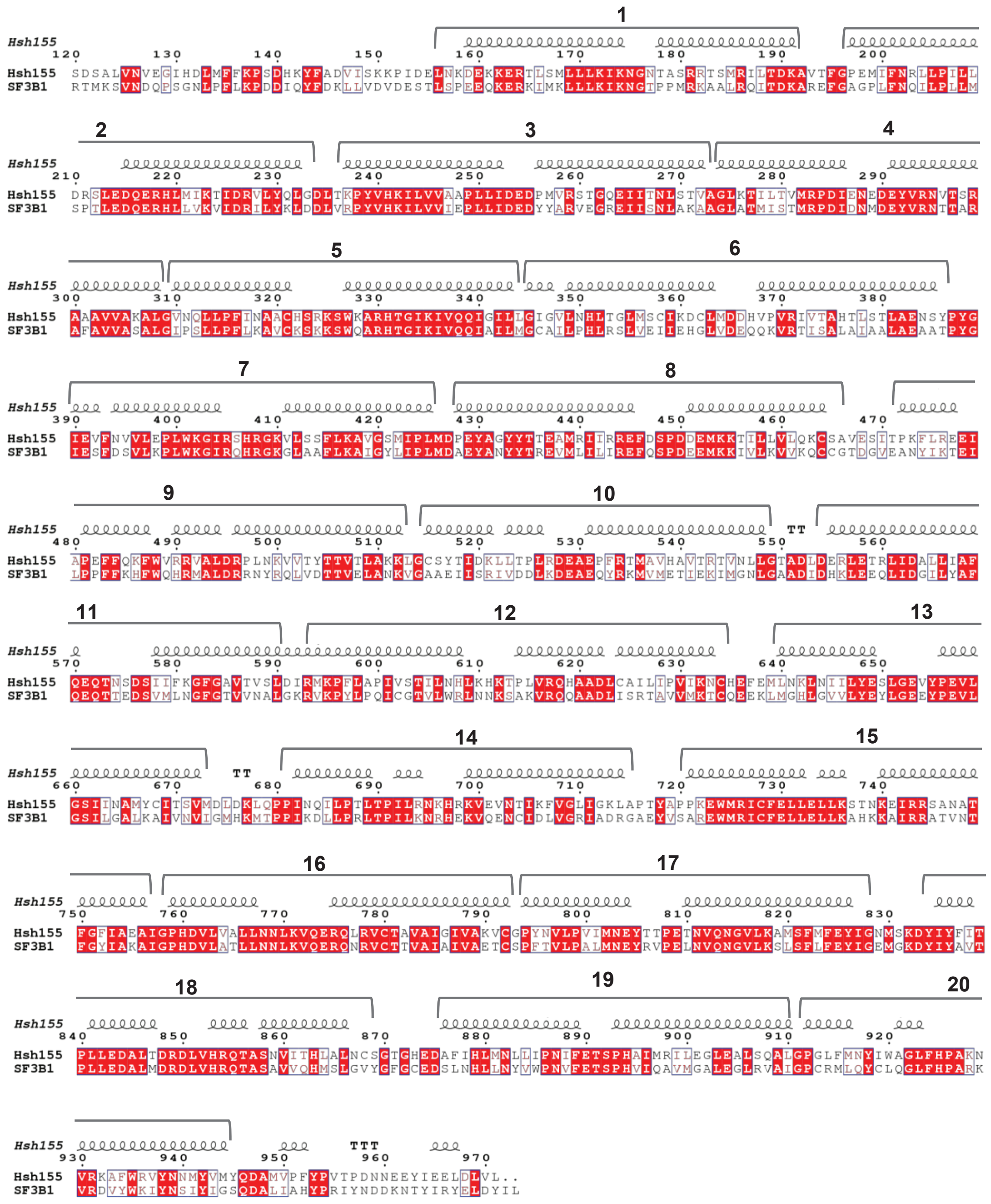

Supplementary Figure 12: Sequence alignment of yeast Hsh155 and human SF3B1.

a

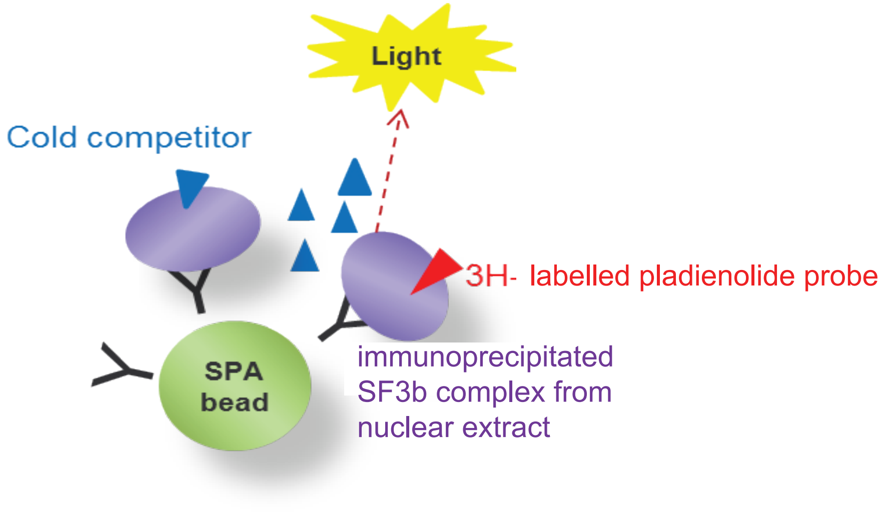

b

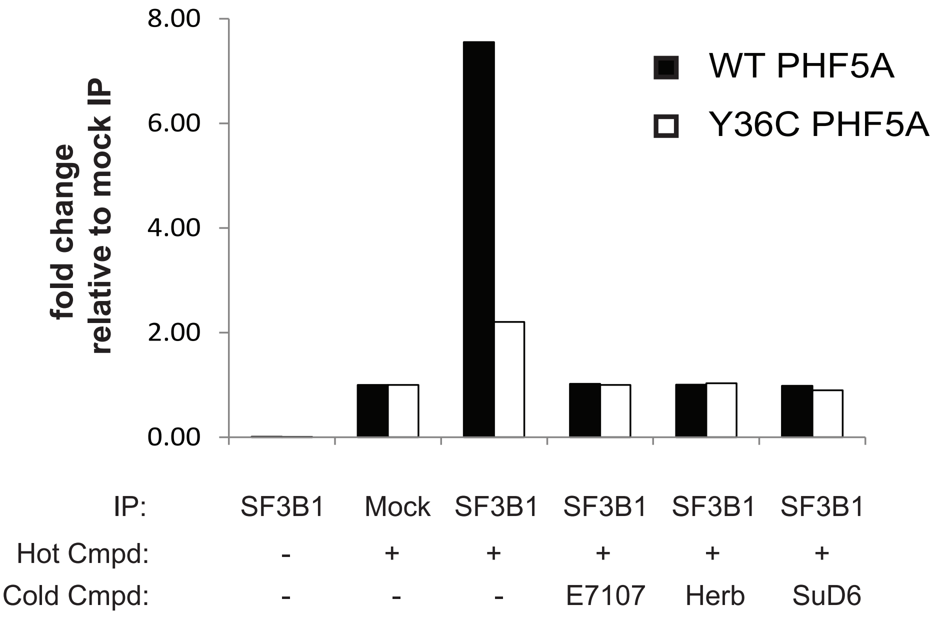

Supplementary Figure 13: PHF5A Y36C reduced splicing modulator's affinity to immunoprecipitated SF3b complex. (a) Scheme of the Scintillation Proximity Assay (SPA). (b) Scintillation Proximity Assay of the ^{3}H-labelled pladienolide analogue (10nM) binding to anti-SF3B1 or mock immunoprecipitated SF3b complex from nuclear extracts containing WT or Y36C PHF5A. Pre-treatment of unlabeled compounds (10μM) were included when indicated.

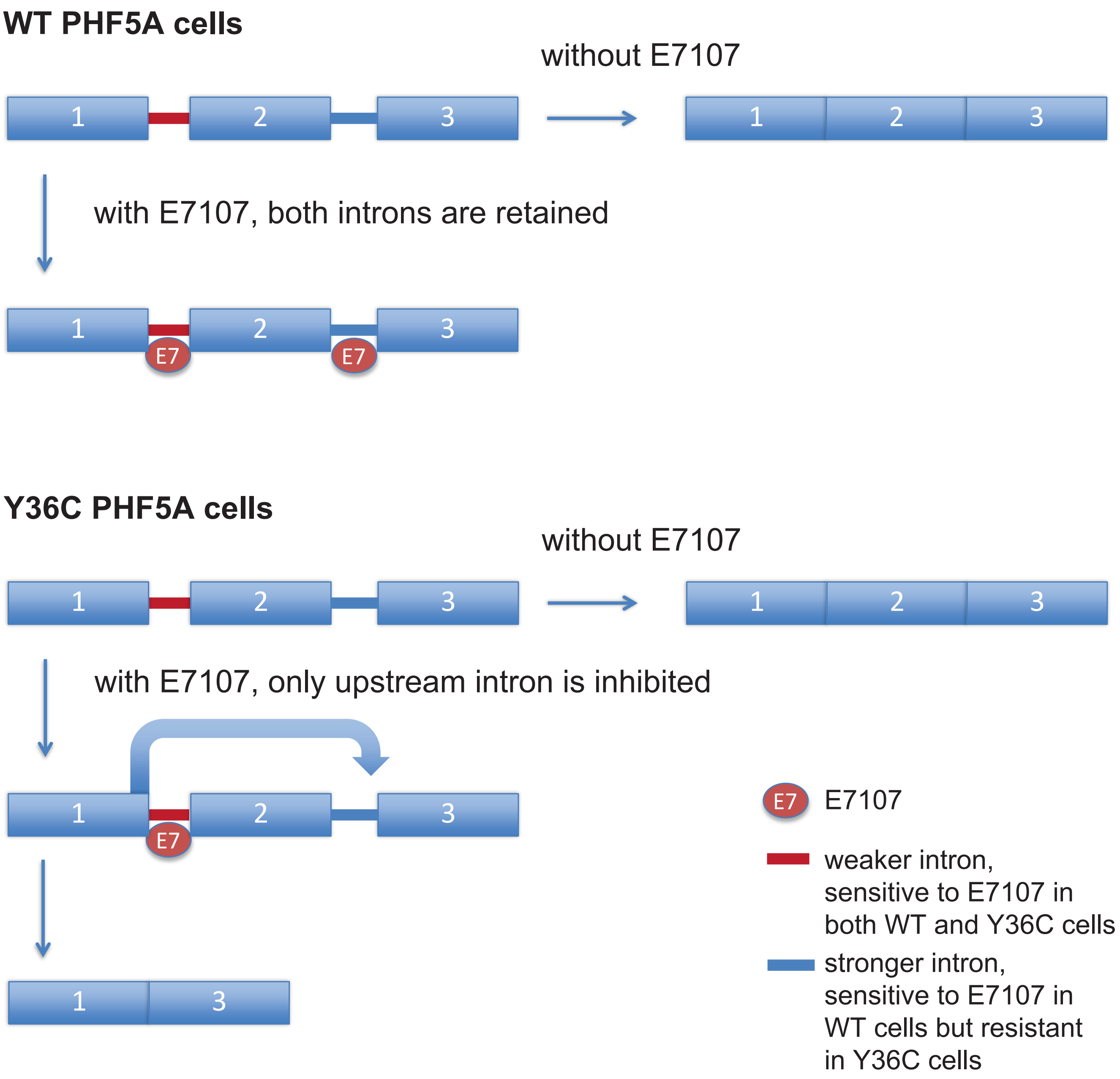

Supplementary Figure 14: Hypothetical model for *MCL1* like “exon - weaker intron - exon- stronger intron - exon” strucutre which underwent IR/ES switch determined by the genotype of PHF5A upon E7107 treatment. In the absence of E7107 treatment, in PHF5A WT and Y36C cells, both introns are recognized by splicesome leading to production of mature mRNAs. In PHF5A WT cells, both introns are sensitive to E7107 leading to IR of both introns. However, in PHF5A Y36C cells as the association of the compound with the PHF5A-SF3B1 interface is reduced, E7107 would become less efficient in the competition with the downstream, stronger intron while maintaining its inhibition with the upstream, weaker intron. Therefore, splicesome would be able to recognize the 3’ splice site of the downstream stronger intron and lead to direct ligation of exon 1 to exon 3.

Figure 2a

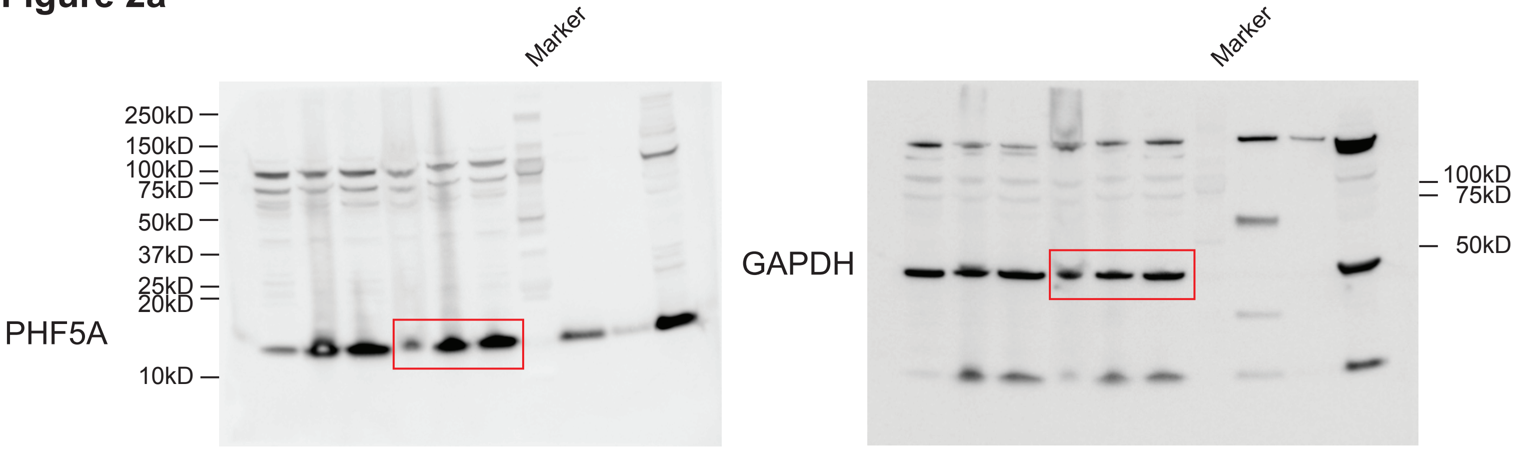

Figure 2c

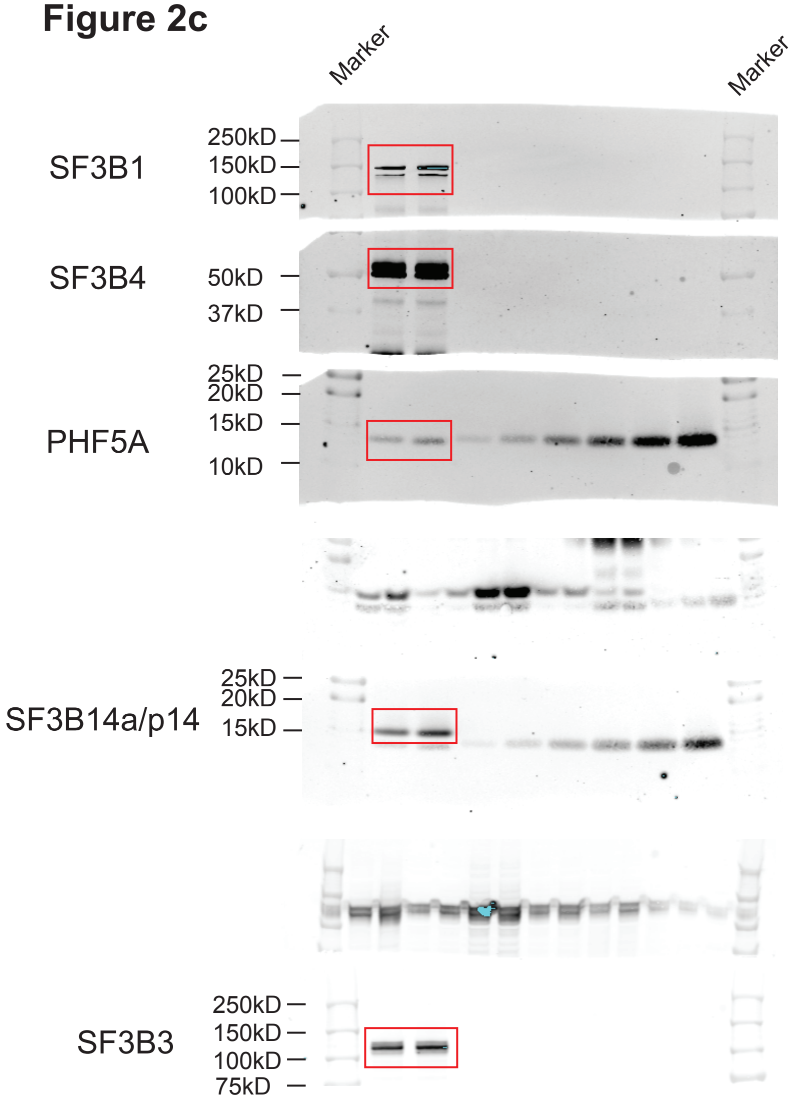

Figure 7a

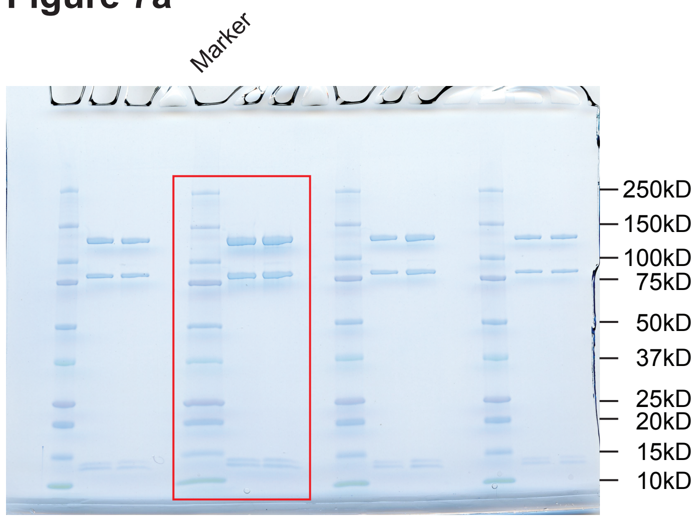

Figure 7e

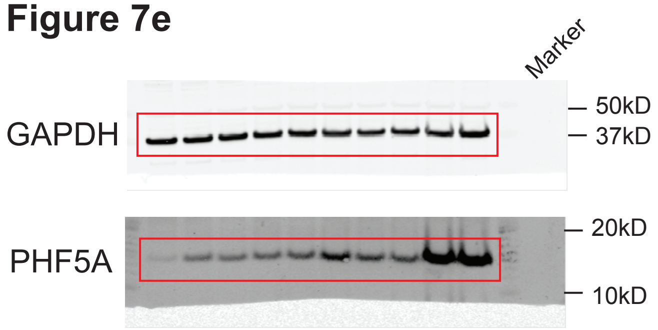

Supplementary Figure 15: Uncropped western blot and gel images. Cropped region shown in indicated main figures are highlighted with red box.

E7107: LC/MS, Purity: 100%

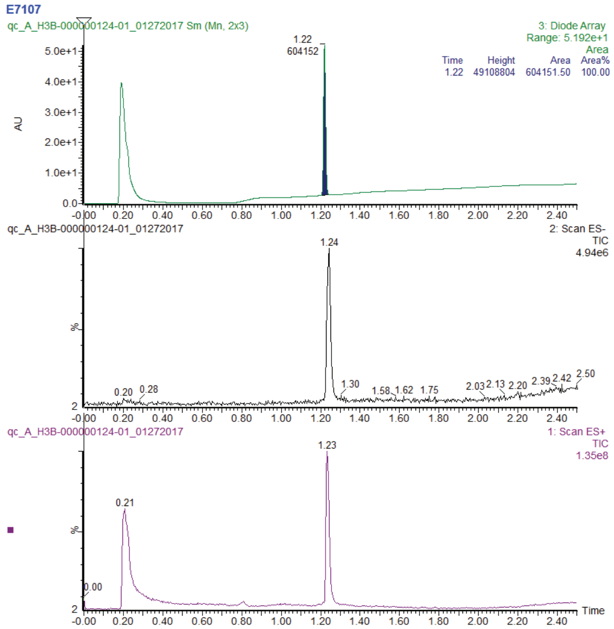

E7107: MS $C_{40}H_{66}N_2O_9$ $(M+H)^+$
Calculated 719.48, Actual 719.74.

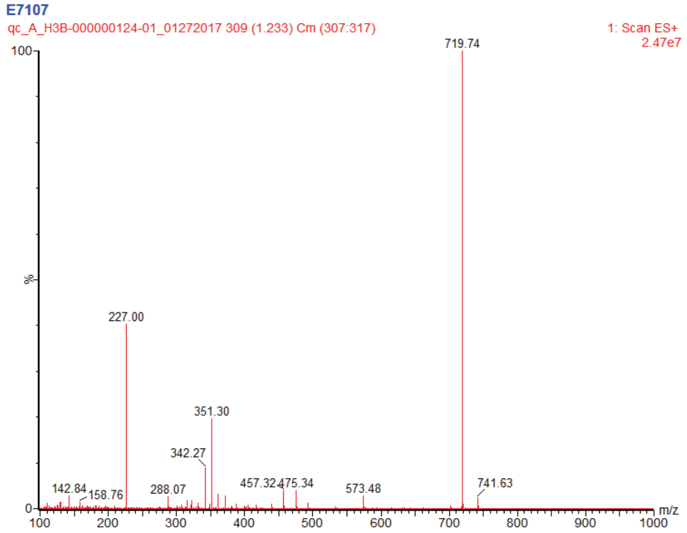

E7107: 1H-NMR

^{1}H NMR (400 MHz, C5D5N) δ 0.82 (d, *J*=6.78 Hz, 3H) 1.04 (t, *J*=7.34 Hz, 3H) 1.13 (d, *J*=6.90 Hz, 3H) 1.22-1.30 (m, 4H) 1.33-1.42 (m, 4H) 1.47-1.56 (m, 3H) 1.54 (s, 3H) 1.59 (s, 3H) 1.59-1.66 (m, 3H) 1.68-1.70 (m, 1H) 1.72-1.75 (m, 1H) 1.73 (s, 3H) 1.78-1.84 (m, 1H) 1.93 (dd, *J*=13.68, 6.53 Hz, 1H) 1.95-1.99 (m, 1H) 2.11 (m,1H) 2.23 (dd, *J*=13.68, 5.14 Hz, 1H) 2.27 (br s, 4H) 2.38 (m, 1H) 2.65 (m, 1H) 2.68 (m, 1H) 2.73 (dd, *J*=14.04, 4.04 Hz, 1H) 3.09 (dd, *J*=7.65, 2.13 Hz, 1H) 3.32-3.37 (m, 1H) 3.57 (br s, 4H) 3.96 (m, 1H) 4.07 (m, 1H) 5.31 (d, *J*=10.67 Hz, 1 H) 5.52 (br s, 1H) 5.55 (d, *J*=9.79 Hz, 1H) 5.85 (br s, 1H) 5.83-5.91 (m, 2H) 6.11 (d, *J*=15.06 Hz, 1H) 6.34 (dd, *J*=15.06, 9.66 Hz, 1H) 6.41 (d, *J*=11.7 Hz, 1H) 6.43 (br s, 1H) 6.95 (dd, *J*=15.12, 11.11 Hz, 1H)

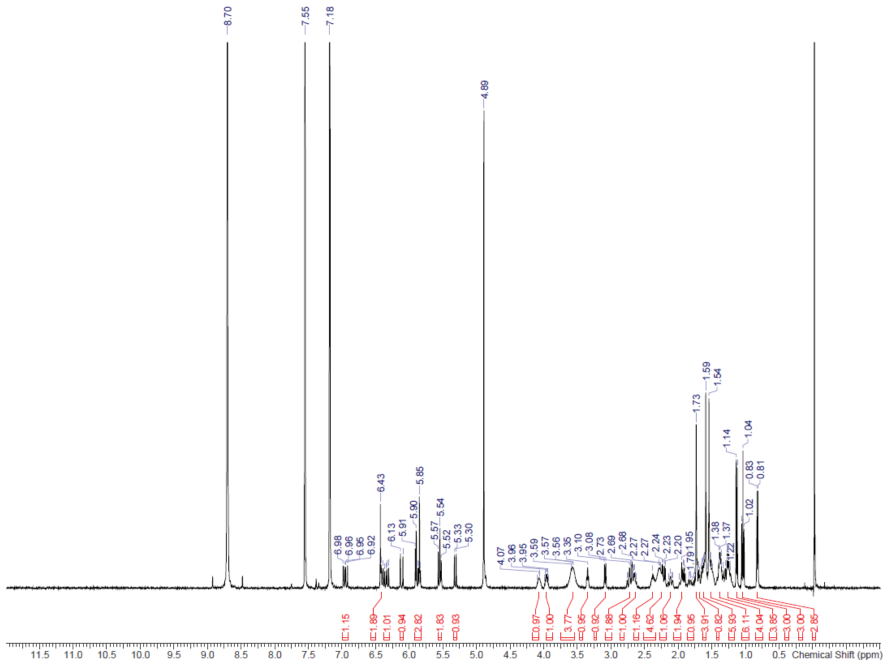

Supplementary Figure 16: Purity and Identity of compounds used in the study. LCMS profile (top) and 1H-NMR spectra (bottom) of E7107 are shown. The first peak in the LCMS at 0.2 min corresponds to the solvent front and is omitted from the integration. The main peak in the mass spec shows the expected mass for the compound. The NMR spectra is annotated with the chemical shifts (blue font) and the number of protons integrated (red font). The large peaks with no integration correspond to solvent peaks.

Herboxidiene: LC/MS, Purity: 100%

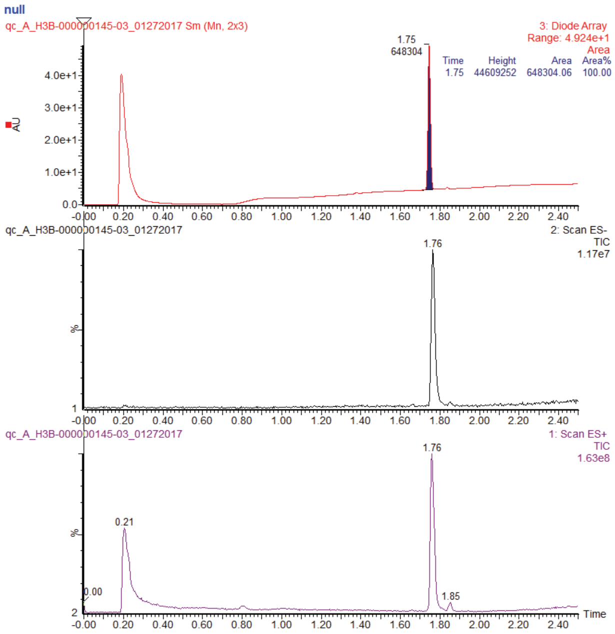

Herboxidiene: MS $C_{25}H_{42}O_6$
Calculated 461.30 $(M+Na)^+$. Actual 461.33

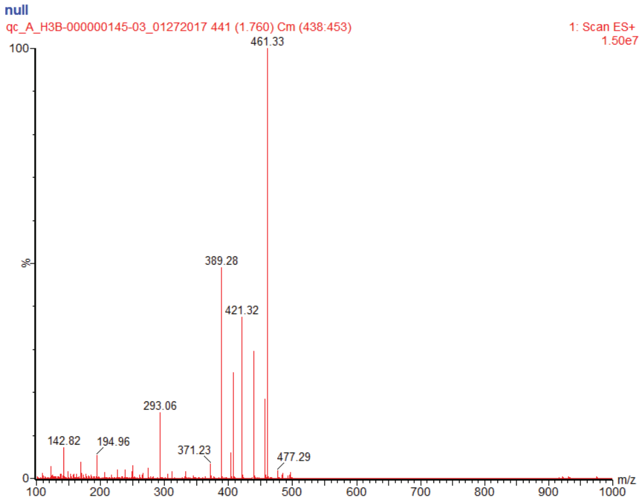

Herboxidiene: 1H-NMR

^{1}H NMR (400 MHz, CD3OD) δ 0.69(d, *J*=6.53 Hz, 3H) 0.84(d, *J*=7.03 Hz, 3H) 1.05(d, *J*=6.65 Hz, 3H) 1.11(d, *J*=6.40 Hz, 3H) 1.14 - 1.27(m, 2H) 1.28(s, 3H) 1.29 - 1.41(m, 1H) 1.45 - 1.60(m, 2H) 1.70(d, *J*=1.13 Hz, 3H) 1.70 - 1.75(m, 1H) 1.82 - 1.90(m, 1H) 1.93(dd, *J*=13.43, 4.27 Hz, 1H) 2.40(dd, *J*=15.31, 5.77 Hz, 1H) 2.42 - 2.51(m, 1H) 2.48(dd, *J*=15.31, 7.28 Hz, 1H) 2.66(d, *J*=9.41 Hz, 1H) 2.98(dd, *J*=6.27, 4.27 Hz, 1H) 3.33 - 3.39(m, 1H) 3.53(s, 3H) 3.72 - 3.85 (m, 2H) 5.48(dd, *J*=15.00, 9.10 Hz, 1H) 5.92(d, *J*=10.79 Hz, 1H) 6.30(dd, *J*=15.06, 10.79 Hz, 1H)

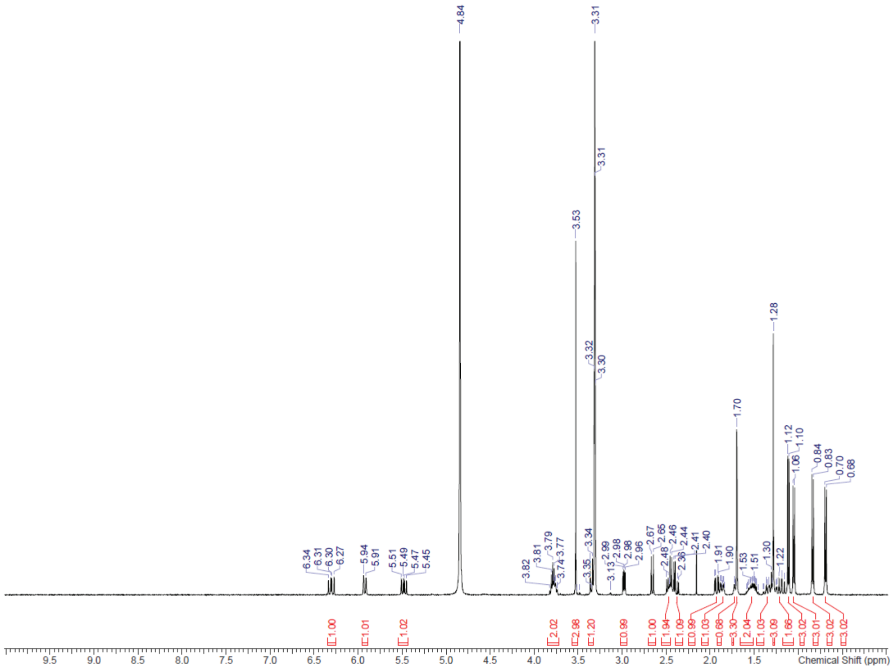

Supplementary Figure 16: Purity and Identity of compounds used in the study (continued). LCMS profile (top) and 1H-NMR spectra (bottom) of Herboxidiene are shown. The first peak in the LCMS at 0.2 min corresponds to the solvent front and is omitted from the integration. The main peak in the mass spec shows the expected mass for the compound. The NMR spectra is annotated with the chemical shifts (blue font) and the number of protons integrated (red font). The large peaks with no integration correspond to solvent peaks.

Spliceostatin A: LC/MS, Purity: 96.4%

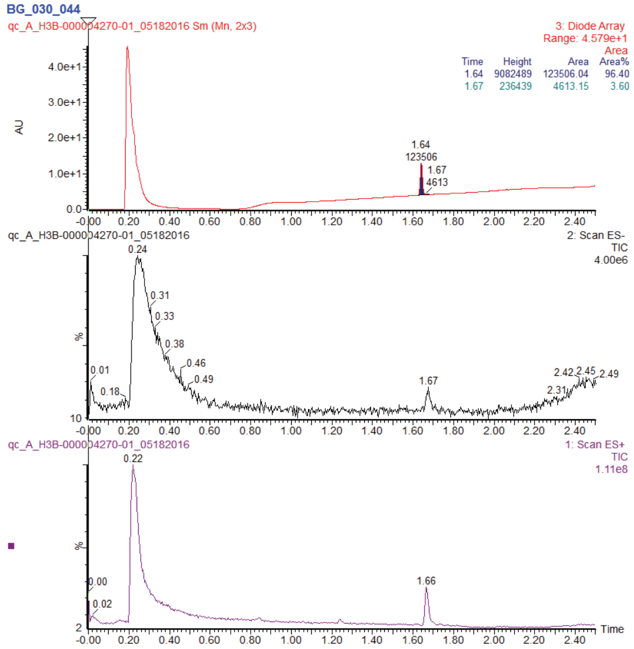

Spliceostatin A: MS $C_{28}H_{43}NO_8$
Calculated 544.30 (M+Na)+. Actual 544.48.

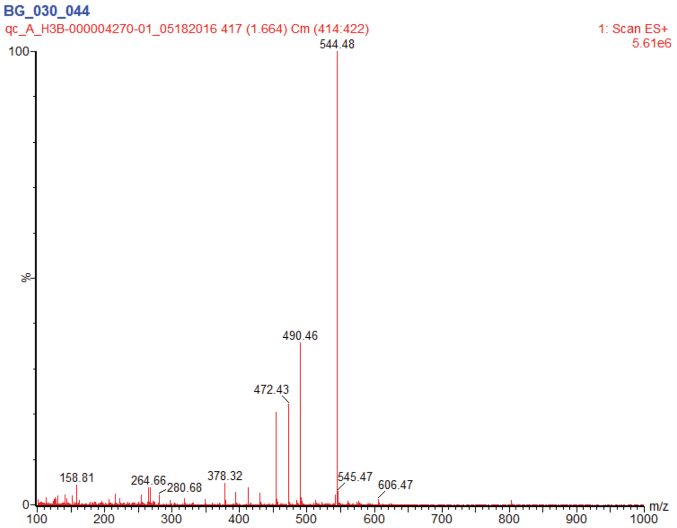

Spliceostatin A: 1H-NMR

^{1}H NMR (400 MHz, CDCl3) δ 1.02 (d, *J*=7.40 Hz, 3H) 1.15 (d, *J*=6.53 Hz, 3H) 1.38-1.40 (m, 6H) 1.68-1.76 (m, 3H) 1.79 (s, 3H) 1.91-1.94 (m, 2H) 2.04 (s, 3 H) 2.18 - 2.43 (m, 3H) 2.49 (d, *J*=4.64 Hz, 1H) 2.99 (d, *J*=4.64 Hz, 1H) 3.28 (s, 3H) 3.50-3.54 (m, 1H) 3.58-3.63 (m, 1H) 3.69-3.64 (m, 1H) 3.91-3.98 (m, 1H) 4.05 (m, 1H) 5.51 (t, *J*=7.03 Hz, 1H) 5.66-5.76 (m, 2H) 5.89 (dd, *J*=11.54, 7.91 Hz, 1H) 5.99 (d, *J*=9.16 Hz, 1H) 6.22-6.30 (m, 1H) 6.40 (d, *J*=15.69 Hz, 1H)

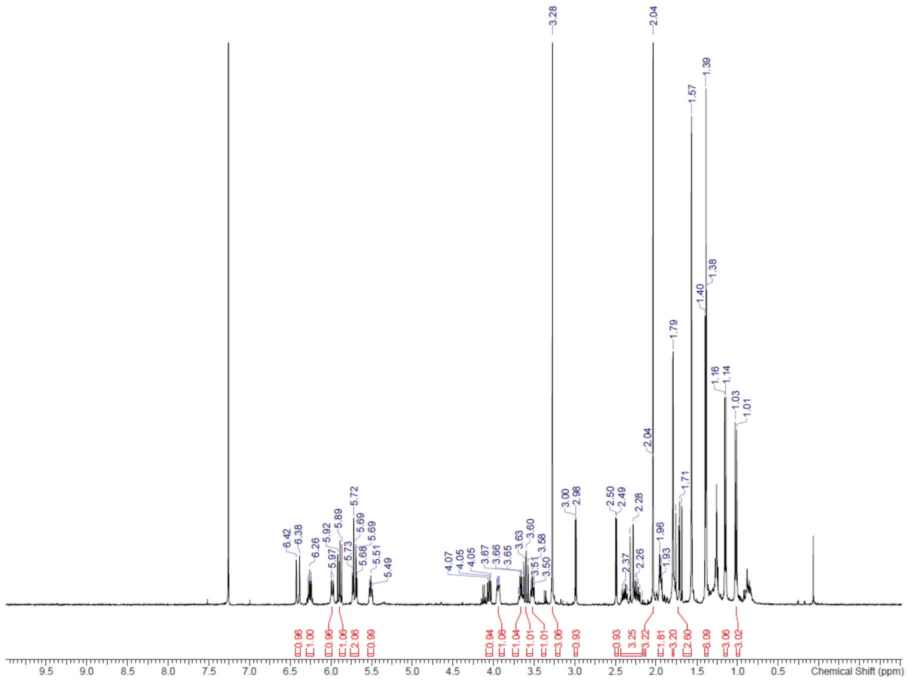

Supplementary Figure 16: Purity and Identity of compounds used in the study (continued). LCMS profile (top) and 1H-NMR spectra (bottom) of Spliceostatin A are shown. The first peak in the LCMS at 0.2 min corresponds to the solvent front and is omitted from the integration. The main peak in the mass spec shows the expected mass for the compound. The NMR spectra is annotated with the chemical shifts (blue font) and the number of protons integrated (red font). The large peaks with no integration correspond to solvent peaks.

Sudemycin D6: LC/MS, Purity: 100%

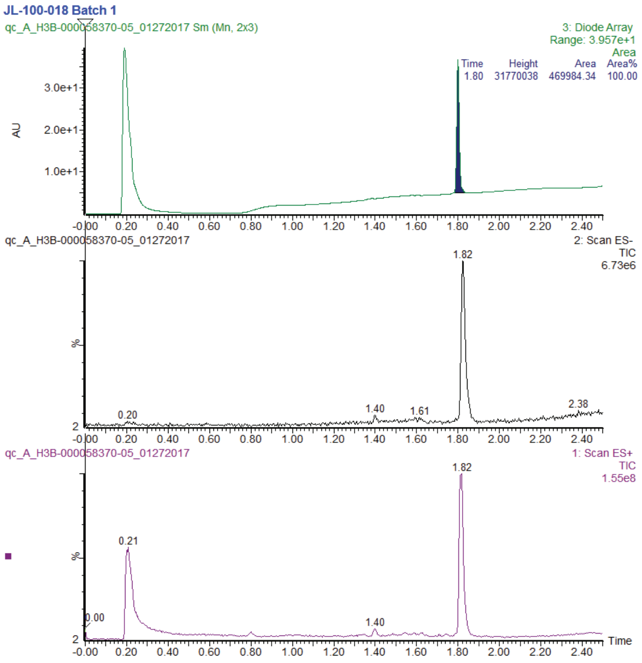

Sudemycin D6: MS $C_{27}H_{42}N_2O_5$
Calculated 475.31 $(M+H)^+$, 497.31 $(M+Na)^+$.
Actual 475.36, 497.40.

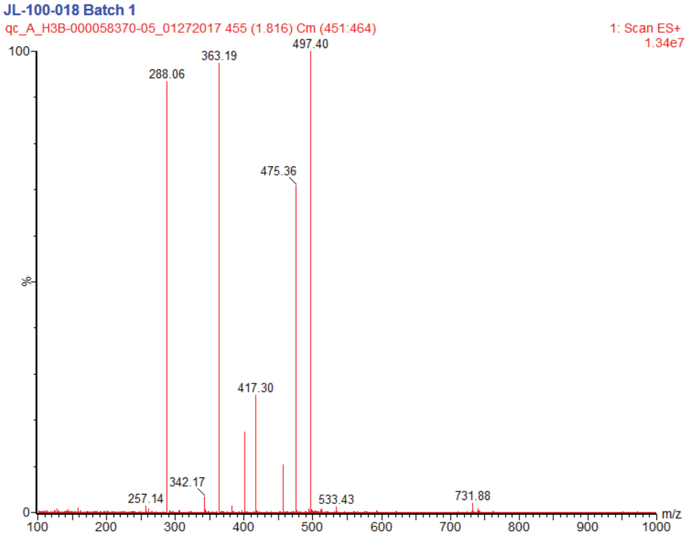

Sudemycin D6: 1H-NMR

^{1}H NMR 1H NMR (400 MHz, CDCl3) δ ppm 1.14-1.24 (m, 2 H) 1.28 (s, 3H) 1.31 (d, J= 5.90H, 3H) 1.35-1.38 (m, 2H) 1.40 (s, 3H) 1.44-1.48 (m, 1H) 1.59-1.63 (m, 4H) 1.6-1.81 (m, 5H) 1.89- 2.02 (m, 2H) 2.10 (br t, *J*=7.09 Hz, 2H) 2.57 (s, 2H) 2.79 (d, *J*=4.89 Hz, 3H) 4.12 (m, 1H) 4.44-4.49 (m, 1 H) 4.74 (m, 1 H) 5.48 - 5.65 (m, 4 H) 5.82 (d, *J*=10.92 Hz, 1H) 6.27 (d, *J*=15.69 Hz, 1H) 7.94 (br d, *J*=7.28 Hz, 1H)

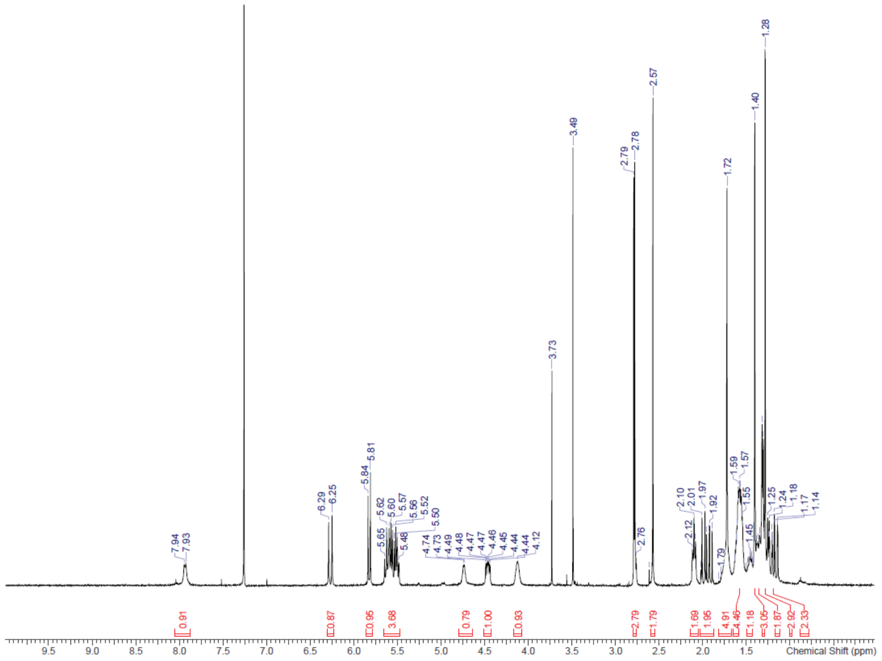

Supplementary Figure 16: Purity and Identity of compounds used in the study (continued). LCMS profile (top) and 1H-NMR spectra (bottom) of Sudemycin D6 are shown. The first peak in the LCMS at 0.2 min corresponds to the solvent front and is omitted from the integration. The main peak in the mass spec shows the expected mass for the compound. The NMR spectra is annotated with the chemical shifts (blue font) and the number of protons integrated (red font). The large peaks with no integration correspond to solvent peaks. Two peaks at delta 3.49 and 3.73 correspond to the two solvents (methanol and 1,2-dichloroethane, respectively) that were used to transfer the material.

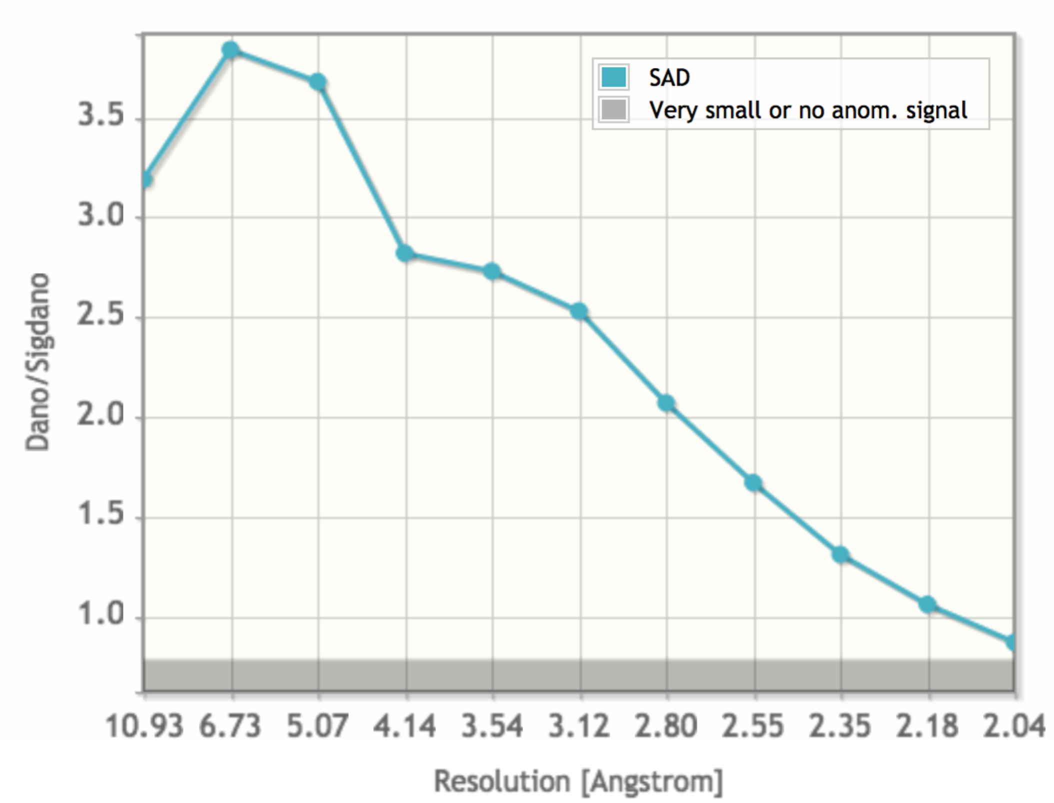

Supplementary Figure 17: Anomalous signal versus resolution. a cutoff for SAD resolution is that Dano/SigDano > 0.5 and a more stringent cutoff would be > 1.2, indicating the anomalous signal for this dataset is 2.04 or 2.35 Å.

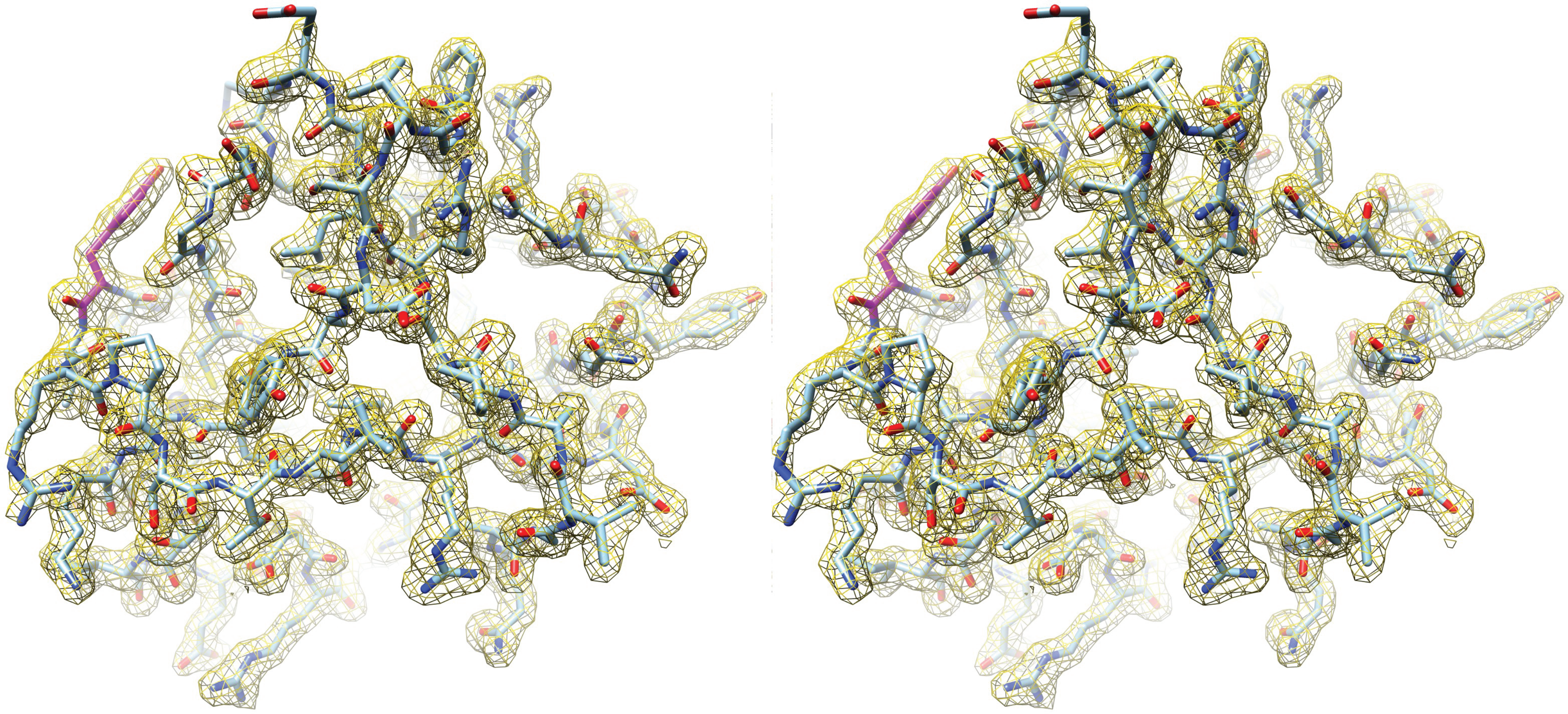

Supplementary Figure 18: Stereo view of PHF5A showing representative electron density from the final refined 2Fo-Fc map contoured at 1sigma. Tyrosine 36 is highlighted in magenta. For clarity, waters were omitted from the map calculation. The figure was generated using the program Chimera.

Supplementary Table 1: Primer and Probe Sequences

Taqman Gene Expression Primer and Probe Sequences

***MCL1-L* probe set**	
Forward Primer	ATATGCCAAACCAGCTCCTAC
Probe	AGAACTCCACAAACCCATCCCAGC
Reverse Primer	AAGGACAAAACGGGACTGG
***MCL1-S* probe set**	
Forward Primer	AAAGCCAATGGGCAGGT
Probe	TCCACAAACCCATCTTGGAAGGCC
Reverse Primer	CCACCTTCTAGGTCCTCTACAT
***MCL1* intron1 probe set**	
Forward Primer	GACAAAGGAGGCCGTGAGGA
Probe	GTTTGTTACGCCGTCGCTGAAA
Reverse Primer	TCAGGCATGCTTCGGAAACTGGA
***MCL1* intron2 probe set**	
Forward Primer	GCCCCGGGGTGAATAATAATTGGTTTACT
Probe	TTTCTAGGATGGGTTTGTGGAGTT
Reverse Primer	CCTGATGCCACCTTCTAGGTCCTCTAC
**pan *MCL1* probe set**	
Forward Primer	GCCAAGGACACAAAGCCAAT
Probe	CTGGAGACCTTACGACGGGTTGGG
Reverse Primer	AAGGCCGTCTCGTGGTT
***SLC25A19* mature form probe set**	Life Technologies Assay ID = Hs00222265_m1
***EIF4A1* pre-mRNA form probe set**	Life Technologies Assay ID = AJRR9DL
**AD2 probe set**	
Forward Primer	ACTCTCTTCCGCATCGCTGT
Probe	CTGTTGGGCTCGCGGTTG

Reverse Primer	CCGACGGGTTTCCGATCCAA
**FTZ probe set**	
Forward Primer	TGGCATCAGATTGCAAAGAC
Probe	CGAAACGCACCCGTCAGACG
Reverse Primer	ACGCCGGGTGATGTATCTAT

Site-Directed Mutagenesis Primer Sequences

**PHF5A Y36C**	
Forward Primer	GTGTGTGATTTGTGACTCCTGTGTGCGTCCCTGCACTCTGG
Reverse Primer	CCAGAGTGCAGGGACGCACACAGGAGTCACAAATCACACAC
**PHF5A Y36A**	
Forward Primer	GAGTGCAGGGACGCACAGCGGAGTCACAAATCACAC
Reverse Primer	GTGTGATTTGTGACTCCGCTGTGCGTCCCTGCACTC
**PHF5A Y36E**	
Forward Primer	AGAGTGCAGGGACGCACCTCGGAGTCACAAATCACAC
Reverse Primer	GTGTGATTTGTGACTCCGAGGTGCGTCCCTGCACTCT
**PHF5A Y36F**	
Forward Primer	AGAGTGCAGGGACGCACGAAGGAGTCACAAATCACAC
Reverse Primer	GTGTGATTTGTGACTCCTTCGTGCGTCCCTGCACTCT
**PHF5A Y36R**	
Forward Primer	GAGTGCAGGGACGCACACGGGAGTCACAAATCACAC
Reverse Primer	GTGTGATTTGTGACTCCCGTGTGCGTCCCTGCACTC
**PHF5A Y36S**	
Forward Primer	CCAGAGTGCAGGGACGCACGCTGGAGTCACAAATCACACAC

Reverse Primer	GTGTGTGATTTGTGACTCCAGCGTGCGTCCCTGCACTCTGG
**PHF5A Y36W**	
Forward Primer	AGAGTGCAGGGACGCACCCAGGAGTCACAAATCACAC
Reverse Primer	GTGTGATTTGTGACTCCTGGGTGCGTCCCTGCACTCT
**PHF5A V37C**	
Forward Primer	CCAGAGTGCAGGGACGGCAATAGGAGTCACAAATCACACACTTG
Reverse Primer	CAAGTGTGTGATTTGTGACTCCTATTGCCGTCCCTGCACTCTGG
